# Education and Licensing of Horse Owners: Addressing Poor Horse Welfare in the UK

**DOI:** 10.3390/ani15071037

**Published:** 2025-04-03

**Authors:** Aurelia Hall-Bromley, Laura Dixon

**Affiliations:** Animal Welfare Centre, The Royal (Dick) School of Veterinary Studies, Easter Bush Campus, The University of Edinburgh, Midlothian EH25 9RG, UK; aureliahall@hotmail.co.uk

**Keywords:** horse welfare, education, licensing, equine

## Abstract

Horse welfare is a subject of ongoing discussion and scientific investigation. Several solutions to combat poor horse welfare have been suggested by existing research; however, each of these has its limitations. In 2022, it was announced that France was introducing new legislation requiring all horse owners to be licensed on completion of particular horse-care education. This development raises an interesting discussion about whether such a framework could be applied in the UK. This systemised literature review analysed the prevalence of horse welfare concerns in the developed world and the UK, their potential causes and the barriers to reducing them, alongside the most often suggested remedial interventions according to stakeholders and the existing literature. This review also analysed the successes and limitations of education and licensing as possible solutions to horse welfare concerns in the UK. The findings indicate that education can increase horse-owner awareness of welfare issues. However, the literature also indicates that education has a limited influence on human behaviour change. There is also limited evidence in the literature that licensing can improve horse-owner awareness or welfare standards within a population. However, promising areas of future investigation were identified, which could lead to improved horse welfare.

## Simple Summary

Horse welfare concerns have been a subject of increasing discussion for decades. The issue of compromised horse welfare in the UK and across the globe is complex as welfare concerns are varied, wide-ranging and nuanced. Several solutions to combat compromised horse welfare have been suggested by existing research; however, each of these has its limitations. In this study, existing literature regarding horse welfare, education and licensing as potential solutions were analysed. Distress behaviour was the most-cited welfare concern in the literature, narrowly followed by health issues and behavioural issues. The citations for causes of poor welfare were dominated by management and training practices. The analysis found that the highest cited barrier to good welfare was a lack of knowledge, followed by a lack of understanding by horse owners. Further field research into horse welfare causes was most commonly suggested as the best step to address welfare concerns, and increased awareness of welfare issues was suggested as the best solution to prevent welfare issues. In terms of education, the most-cited positive outcomes were increased knowledge, awareness or understanding. However, the most prominent limitation of education was an unclear effect on behaviour as well as other notable factors, such as availability. The most-cited licensing success was used as a consumer tool to aid decision making; however, licensing was limited by enforcement. Taking the relevant literature as a whole, there is no single evident solution that can solve the horse welfare problem; however, there are areas identified that merit closer consideration.

## 1. Introduction

The role of the horse in human society has evolved over the years, with a shift in recent decades towards the position of sporting or companion animals in the developed world, including the United Kingdom [1,2,3]. Any use of horses includes a range of activities and interventions that can, collectively, compromise welfare [4,5]. Domestication itself has been identified as a source of welfare issues, for example, through restriction of movement, social interaction and equine behaviour patterns [1]. Subsequently, recreational horse welfare has become a prominent global concern due to the lack of regulation, as well as the increased diversity of horses and owners, leaving more room for knowledge gaps [6]. Additionally, the popularity of equestrian sport has placed horse welfare under increased scrutiny [7], with ongoing work to reduce risk to an acceptable level [8]. Animal science has also advanced, with welfare science developing rapidly alongside the evolution of the concept of welfare itself [9]. Finally, there has been an emergence of equitation science, which enables us to reconsider horse training practices using learning theory, physics and ethology, with the goal of achieving optimal training aims without compromising welfare through fear or pain [10].

Despite the history of discussion and research demonstrating avoidable risks to the welfare of horses and methods for prevention, recent findings indicate that there remains a high prevalence of welfare problems for horses [11] caused by certain training techniques [1] and husbandry practices [7]. For purposes of this review, the term “welfare” is defined as an individual’s long-term physical and mental state and perception of their situation [12], with consideration of the needs of animals, as stated by the Animal Welfare Act 2006, and their interplay according to the Five Domains Model [13]. For the United Kingdom (UK), estimates of the size of the horse population vary. DEFRA estimated 150,000 horses—in commercial holdings—in 2021, the UN estimated 425,000 in 2019 [14], and the British Equestrian Trade Association (BETA) estimated 847,000 in the same year [15]. BETA also estimated 374,000 horse-owning households in Britain. The limited estimate for the UK horse population is a concern in itself despite requirements for equine passports and microchipping; however, these figures serve to demonstrate the scale of the issues at hand. Almost 850,000 horses are potentially at risk of welfare compromise, with less than half as many owners to take responsibility. Due to the number of privately owned horses and lack of any tailored government legislation specifically for their ownership or management, this review focused on their welfare, with horses involved in the reviewed research being predominantly recreational, sporting or companion animals, as opposed to commercially owned or managed horses.

In 2022, new legislation was introduced in France to make the education of horse owners mandatory through licensing [16]. Although it is too early to assess the success of this legislation, this raises a thought-provoking question. Could education and licensing of horse owners improve the welfare status of the UK horse population? The improvement of welfare issues for UK horses has been described as a relentless challenge due to the complexity of the issue and public perception [17]. Monitoring and reporting also appear to be an issue; not only is the size of the population poorly estimated, but there is a limited overview of the population’s welfare status [18], although attempts have been made to assess the prevalence of specific concerns [18,19,20]. To determine if education and licensing could be a potential UK solution, first, the scale of horse welfare concerns needs to be determined, the likely causes for poor welfare identified, the existing suggestions for improvement explored and the possible applications and limitations of education and licensing assessed.

Additionally, in order to identify a possible successful solution to compromised horse welfare on a national scale, we need to understand the priorities and values held by key stakeholders [21]. For recreational horses, welfare is primarily the responsibility of the owner [7], making the owner the primary stakeholder. Many recreational horse owners have little or no previous knowledge of horse needs [1]. The need for improvement of horse-owner knowledge has been echoed in relation to a variety of welfare concerns [2,22,23,24]. Also, studies have found horse welfare can be impacted by different facets of the horse–human relationship [25,26,27]. Research aimed at understanding stakeholders’ perceptions of horse welfare has been limited [28], as has research into horse–owner relationships [7]. However, several common themes have been identified as factors contributing towards poor horse welfare: accepted social norms, ignorance, indifference, financial constraints and indolence [18,24,29]. Each suggested factor can fall under capability (C), opportunity (O) or motivation (M); the suggested processes underlying human behaviour (B) in the COM-B model [30], which can be targeted to achieve effective behaviour change [31]. Achievable human behaviour changes need to be considered for effective legislation to be developed, followed and enforced.

The aim of this review was to determine if structured education and owner-specific legislation could be a potential solution to common welfare issues of privately owned horses. To meet this aim, the review distilled the most studied welfare concerns relevant to the privately owned UK horse population within the existing research literature by assessing the frequency of research into different welfare issues across the developed world from countries with similar social, political and economic status to the UK and comparing the findings with existing research focussed on the UK. The reasons behind continued welfare issues were also assessed through analysis of poor welfare causes and barriers to good welfare and these were also considered alongside successes and limitations of education use. Finally, the review considered licensing as an enforcer of education and a regulatory tool for horse ownership and welfare issues by assessing and summarising the cited successes and limitations of legislation within the existing literature.

## 2. Materials and Methods

This study was a systemised literature review using the principles of a systematic literature review on a smaller scale. The data were collected between January and March 2023, and the findings assessed between March and July 2023. The review was guided by the PRISMA protocol [32] to enable accurate reporting [33]; the recently updated reporting tool was used [34]. See Appendix A for the completed protocol for this project. The PRISMA checklist was used to ensure that the review had clear aims and objectives and a planned methodology prior to data collection. Any amendments to the methodology are specified, as well as the elements of a full systematic review, which were not possible in this study due to the limited time frame.

The review used two discrete searches to encompass both the horse welfare literature (Search 1) and the literature exploring education and licensing in relation to animal welfare (Search 2). The searches were run first in Web of Science and cross-checked in Google Scholar for comprehensive coverage.

Both searches were limited to the date range of 2006–2023, from the latest update to the Animal Welfare Act, which altered the standards of and obligation to welfare for horse owners [35,36], until the start date of data collection. The literature had to be cited at least once a year on average since publication, i.e., ten times in ten years or twice in two years. This criterion reduced time spent critically analysing papers that were not taken up by continuing research in the field, which may indicate less robust or practically applicable studies or may have been superseded by newer research, thus maintaining the scope of the study within the available timeframe. Both searches excluded literature focussed on developing or least developed countries according to the World Trade Organisation [37] and the classification by the United Nations [38] in order to obtain results most relevant to the UK’s social, economic and political climate. Search 1 was also subject to the following additional exclusion criteria: settings not relevant to private ownerships (for example, veterinary procedures, research, breeding, racing, meat production and industrial operations). Additionally, papers exploring new and alternative methods for assessing welfare were excluded, as their focus was on practical welfare indicators rather than the concerns themselves or accompanying factors. Search 2 also excluded literature focussed on veterinarians or with an industrial focus, as research in these areas was not relevant to licensing or educating the general population.

Table 1 provides details on the search terms applied. Both search terms yielded several thousand results in Web of Science. An additional criterion was added to the results to include “animal welfare” in the topic (title, abstract and keywords). To condense the sample down to the most relevant items and for them to be assessable within the scope of this project, further exclusions were applied. Specifically, the searches were then limited to articles, review articles and entries in English (the majority of results). Search 2 also excluded papers with citation topics of veterinary sciences, nursing and marine biology to automatically exclude papers according to the predetermined criteria. All remaining papers were recorded and checked for potential relevance to the data set, and any not meeting the criteria were manually excluded. Google Scholar did not allow for as detailed filtering of results as the Web of Science. To enable a cross-check within the scope of this review, this search was limited to essential keywords within the title, using the Advanced Search function.

Every paper that met the inclusion criteria was analysed for the validity, repeatability and generalisability of the methods and results. For each paper, the type of methodology and the sample size were recorded, with comments on any potential for bias and observations of the robustness of the methods and quality of the sample.

Descriptive statistics were employed to analyse the data and enable inferences for wider discussion [39]. Frequency distribution and mode were of primary interest, as they would identify which topics were most and least researched or discussed in the literature. While this methodology does mean that the scale of welfare concerns is somewhat reliant on what is easiest or most popular to research, the frequency of papers under each category still served to provide a general picture of the concerns faced in the 17 years preceding this study. Responses were collated under several themes. Search 1 was analysed for details relating to welfare concerns, barriers to good welfare, causes of poor welfare, and suggested solutions/next steps. Search 2 was analysed for details relating to successes of licensing, successes of education, limitations of licensing, and limitations of education. Each paper within a search could contribute multiple inputs across the available themes; for example, in Search 1, a paper could discuss multiple welfare concerns, such as pain, distress and coercive control, while also identifying possible causes, such as training practices and competition. The total frequency of inputs under each theme was also recorded. Measuring the frequency of welfare concerns within the literature could not accurately determine the prevalence or ranking of those concerns for the UK horse population. However, the results were searched for studies that assessed the prevalence, priority, causes and solutions of horse welfare concerns in the UK, and their results are compared to this study’s findings. This can illustrate whether research is currently reflecting the perceived issues in the sector and, thus, whether conclusions from the research are more likely to be applicable to welfare improvement strategies.

## 3. Results

Overall, both searches yielded more applicable papers than anticipated, generating a wealth of data across many themes. It should be noted that the themes for both education and licensing successes/limitations include both influential and outcome factors; for example, Education Successes include parameters that have been argued to make education successful and outcomes that are assessed as a beneficial result of education. Table 2 summarises key findings, the number of papers that fell under each theme (input frequency) and the number of different categories identified within each theme (input variation). Appendix B defines each theme and input in the context of this study, while Appendix C contains the full list of included papers.

A variety of methods and samples were present in both data sets. However, a common theme, with few exceptions, was a gender skew in studies involving human participants. Generally, these studies had more female participants than male, some with as much as an 85:15% split, respectively. This limitation of the literature with a view to informing future action will be discussed further. Studies in Search 1 had numerically smaller sample sizes, on average, than those in Search 2. This is likely due to the larger number of studies involving animals where very large sample sizes can be difficult to obtain, as opposed to sample sizes involving human participants, as in Search 1., which should not affect the validity across the two data sets.

### 3.1. Search 1

Search 1 produced 933 results (413 from Web of Science, 520 from Google Scholar), yielding 124 included papers (89 from Web of Science, 35 additional from Google Scholar). These included 43 Quantitative Experimental studies, 18 Qualitative (interviews, focus groups, etc.), 28 Surveys (quantitative data) and 35 Literature Reviews. Many of the excluded papers were focussing on developing welfare assessments, as well as a small number of studies from developing countries.

### 3.2. Search 2

Search 2 produced 605 results (455 from Web of Science, 150 from Google Scholar), yielding 135 included papers (130 from Web of Science, five additional from Google Scholar). These included 15 Quantitative studies, 27 Qualitative, 68 Surveys and 25 Literature Reviews. Of these, eight were also included in Search 1. Most excluded papers were due to a veterinary or industrial focus.

### 3.3. Descriptive Statistics

The frequency distribution for each theme is presented in Figure 1, Figure 2, Figure 3, Figure 4, Figure 5, Figure 6, Figure 7 and Figure 8. The modal result for each theme is presented in Table 3.

**Figure 1:**
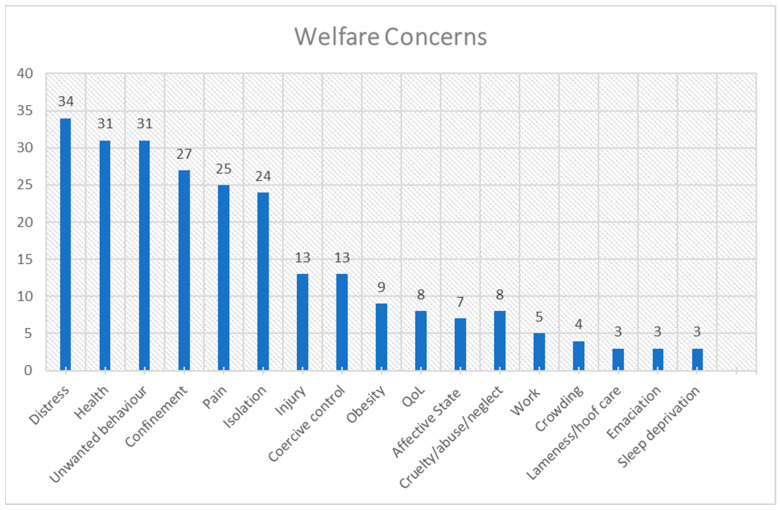
Frequency distribution of welfare concerns (theme 1) from the assessed literature. (QoL = Quality of Life) (Crowding refers to overstocking or too many horses in a given space).

Distress (including stress and fear) was the most-cited welfare concern in the literature, narrowly followed by health issues (including disease) and unwanted behaviours (including stereotypic, aggressive and hyperactive behaviours) (Figure 1). Also highly cited were confinement, pain and isolation (Figure 1).

**Figure 2:**
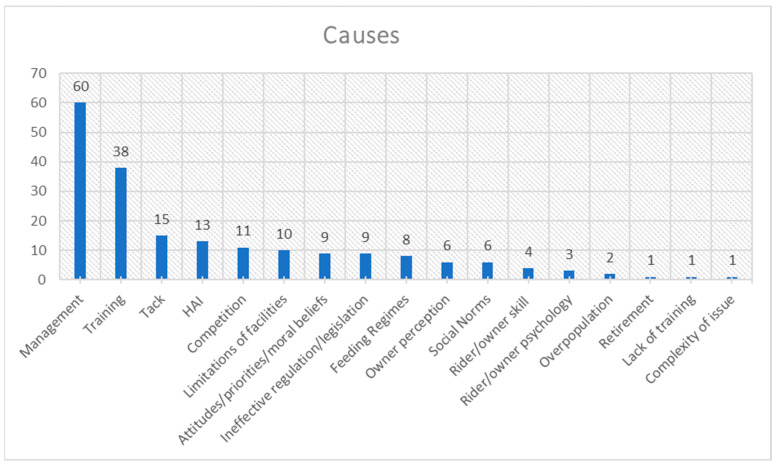
Frequency distribution of causes of poor welfare (theme 2) from the assessed literature. (HAI = Human–Animal Interaction) (Overpopulation refers to too many horses for a given space and can lead to the welfare concern about crowding).

**Figure 3:**
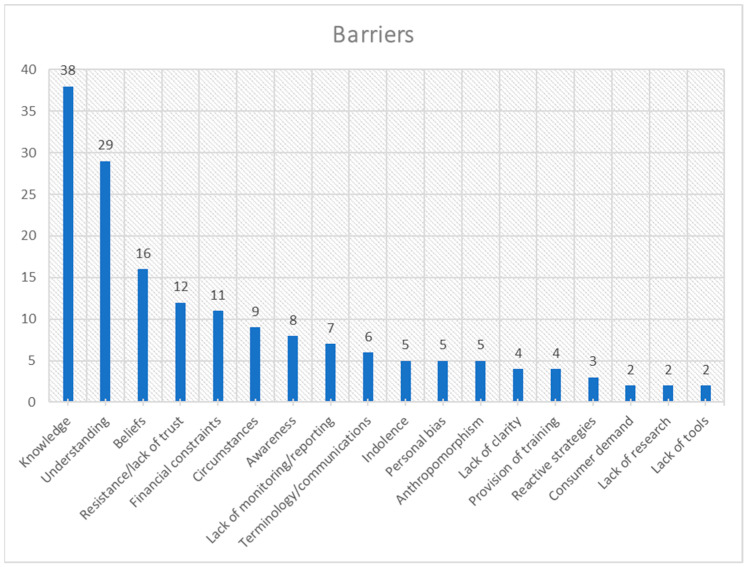
Frequency distribution of barriers to good welfare (theme 3) from the assessed literature. (Indolence refers to a lack of motivation or ‘laziness’ of horse owners to improve equine care knowledge).

The cited causes of poor welfare were dominated by management practices and, to a lesser extent, training practices (Figure 2). There are other notable causes, which will be discussed in more detail. However, many of these can also be related to these two dominant causes; for example, Human–Animal Interactions (HAIs) are intrinsically part of most management and training practices. The highest cited barrier to good welfare was a lack of knowledge, followed by a lack of understanding, of available information about horses’ needs and personal beliefs (Figure 3).

**Figure 4:**
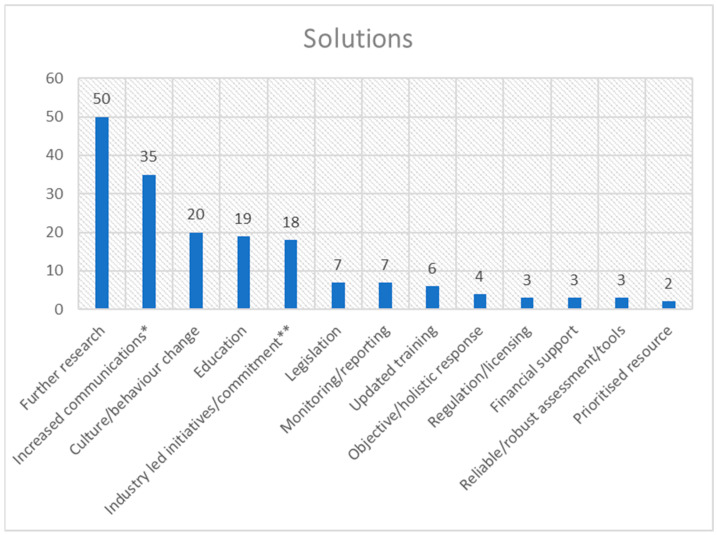
Frequency distribution of solutions to welfare concerns (theme 4) from the assessed literature. [* includes increased awareness. ** includes reviews, example setting, collaboration].

The most-cited solution/next step to improve horse welfare was further research; this was another dominant theme alongside increased communications and awareness (Figure 4). However, three other themes stood out in the data set, albeit to a lesser extent: culture/behaviour change, education and industry-led initiatives (Figure 4).

**Figure 5:**
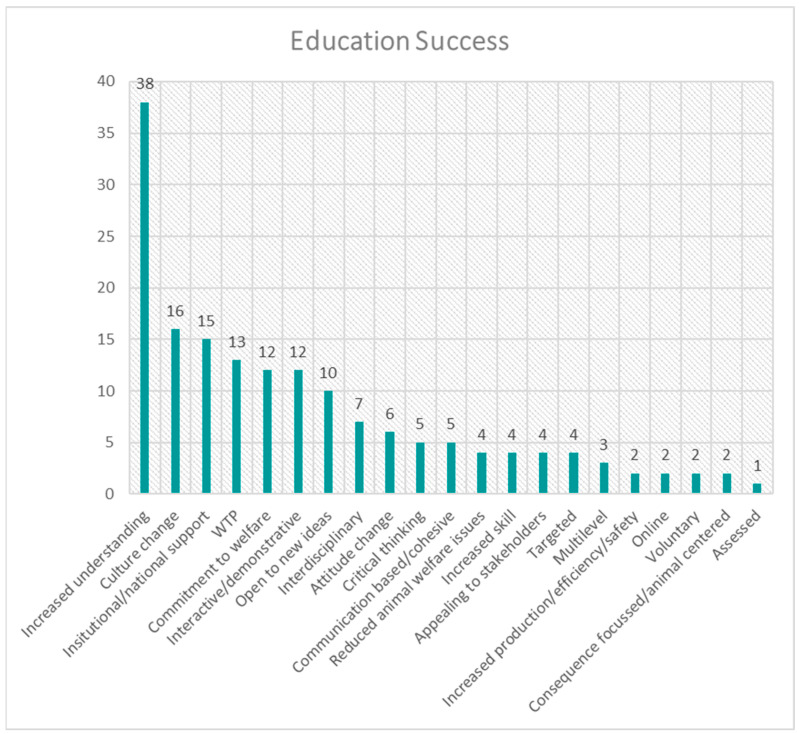
Frequency distribution of successes of education (theme 5) from the assessed literature. (WTP = Willingness/ability To Pay).

**Figure 6:**
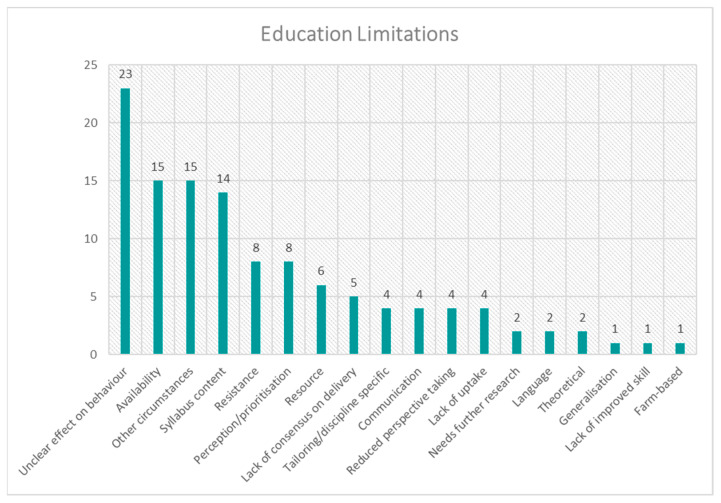
Frequency distribution of limitations of education (theme 6) from the assessed literature.

The successes of education were another data set dominated by increasing understanding, knowledge or awareness, with the secondary success of education indicated as aiding cultural change. One of the highest cited factors for education to be successful was institutional/national support (Figure 5); the possible interplay of this with the suggested solution of industry-led commitment (Figure 4) will be discussed in more detail later. The cited limitations of education were most prominently an unclear effect on behaviour (human); however, availability, other circumstances and syllabus content were also notable (Figure 6).

**Figure 7:**
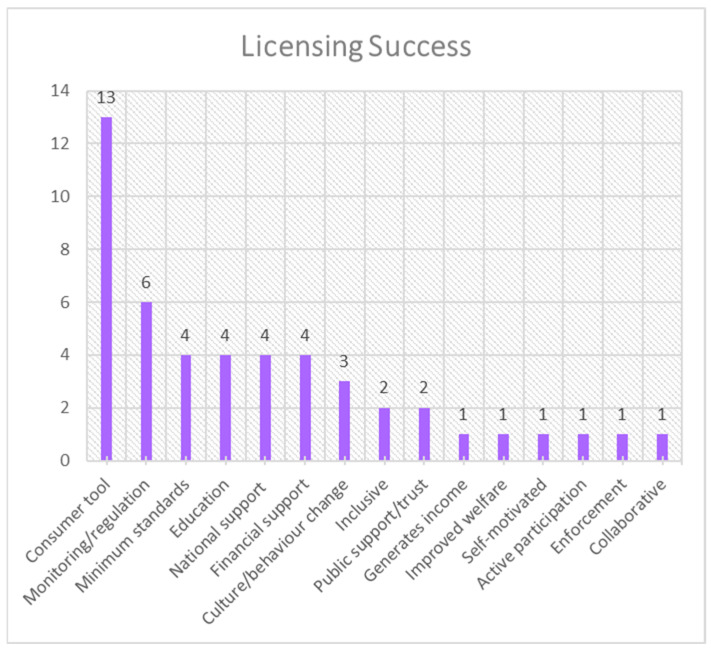
Frequency distribution of successes of licensing (theme 7) from the assessed literature.

**Figure 8:**
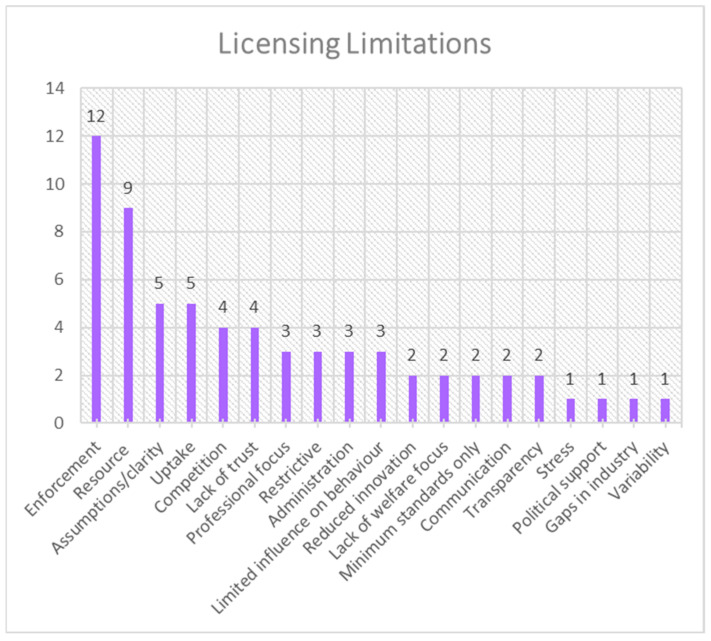
Frequency distribution of limitations of licensing (theme 8) from the assessed literature. (Resource refers to the availability of a program, plan, software, etc., that can be used for licensing.) (Restrictive refers to the limitations or perceptions of limitations the license may place on people’s practices and can lead to resistance to the license.).

There were less data present in the data set for licensing successes and limitations. However, from the available inputs, the most-cited success of licensing was as a consumer tool—e.g., license information in packaging to inform consumers aid decision making (Figure 7)—and the most-cited limitation was enforcement, followed closely by available resource for implementation (Figure 8). There are other moderately cited factors, such as monitoring and regulation as a success of licensing (Figure 7), which will be discussed further.

## 4. Discussion

The variety of welfare concerns cited in the literature (Table 2, Figure 1) emphasises the complexity of welfare as an issue. The results of this review will be assessed in relation to the prevalence of welfare concerns and their corresponding causes. Additionally, reasons for and against education and licensing as a method to improve privately owned horse welfare will be evaluated with consideration of the cited barriers to improvement.

Although further research is the most suggested next step to address horse welfare concerns in the assessed literature (Table 3), this discussion is primarily focused on how existing knowledge could be used to combat horse welfare issues now, which will make up the majority of the evaluation. There are undoubtedly areas where further research is needed, and those that are key to this study’s findings will be discussed. However, in general, more research itself will not necessarily provide solutions and may actually lead to more questions or areas of further research. This is still valuable; however, is less likely to be directly applicable in practical scenarios that private horse owners can implement

### 4.1. Welfare Concerns and Causes

Within the assessed literature, few papers have also sought to prioritise or rank horse welfare concerns in particular countries (e.g., [18,40,41]). Rioja-Lang et al. and Horseman et al. surveyed equine experts and/or stakeholders to determine UK horse welfare priorities. Overall, the highest-ranked concerns by Rioja-Lang et al. [41] and Horseman et al. [18] can be linked back to management practices that would appear to support the initial findings of this study (Figure 2). The differences between this study and those consultations and interviews could be for assorted reasons. Firstly, Rioja-Lang et al. [41] explored a consensus for a range of species as well as horses; thus, the sample size for horse welfare data was only 19 of the 117 experts consulted, compared with a sample of 31 by Horseman et al. [18], whereas this study included contributions from 124 papers. Although, the Horseman et al. [18] study used an opportunistic sampling technique, the data collected may not be as comprehensive as the more structured approach by Rioja Lang et al. [41]. Equally, the focused data collection of interviews and focus groups cannot be directly compared to the findings of a broad literature review. Secondly, the Rioja Lang et al. study [41] was only seeking expert opinions, primarily researchers, charity workers and industrial representatives, with small representation from vets or government workers. By contrast, the data set for this study is based on a wide range of research, including opinions from owners and vets. Thirdly, the study by Rioja-Lang et al. included concerns that were excluded from this study, such as euthanasia decisions and breeding. Finally, the Rioja-Lang et al. [41] study categorised some concerns differently. For example, fear/distress/injury resulting from specific practices compared to stress/distress/fear being one category and work, training, health concerns and pain being separate categories. Similarly “lack of recognition of pain behaviour” versus pain itself as a category and “large worm burdens” as a specific category that would be encompassed within health in this study. On the other hand, obesity is categorised the same in both studies, and some terms are very similar, such as “unsuitable diets” and feeding regimes, and “poorly fitting and restrictive tack” compared to simply tack (including bit use). The former study also limited the rankings to the top ten, whereas this study has counted all cited welfare issues. However, the similarity of results both in Rioja-Lang et al. [41] and Horseman et al. [18] concurs with this study’s findings, as management practices are the most-cited cause of poor welfare (Table 3). Management practices encompass some of the most-cited welfare concerns, for example health issues (e.g., [42]), and confinement and isolation (Figure 1) (e.g., [43,44]). Management practices can also include feeding regimes that themselves ranked within the top ten causes of welfare concerns (Figure 2) (e.g., [45]). As the horse owners for privately owned horses are mainly responsible for these practices [46,47], interventions aimed at private horse owners have the potential to improve welfare for a large population of horses.

Training practices are another area of concern, according to the literature (Figure 2). For example, it has been argued that lungeing, or round-pen training, poses a significant welfare concern without proper application of learning theory [23]. Horses may perceive themselves as being chased without a means of escape as opposed to being at liberty to respond to training stimuli [23]. This could lead to ‘learned helplessness’, whereby horses learn that movements to escape a negative situation are not possible, leading to poor welfare outcomes [11,48]. Equally, horses can fail to learn established boundaries or engage with training stimuli if these practices are undertaken improperly [9]. Training practices can also be linked to other highly cited welfare concerns of distress, unwanted behaviour, pain, injury and coercive control (Figure 1). The existing literature suggests a number of reasons that training practices may be a source of welfare concerns, even putting aside intentionally harsh practices that have been largely outlawed. Training practices may be hindered by misleading terminology that is poorly understood [49]. Equally, training may be limited by practices developed prior to recent findings. For example, research into horse cognition has yielded results that contradict commonly held beliefs about horse behaviour [50], including the formation of stereotypic behaviours in relation to dopamine regulation. These discoveries also, in turn, encourage reflection on common training and management practices, such as positive versus negative reinforcement learning techniques and the still widespread practice of long-term stabling (confinement) [51]. As a result, understanding horse learning can benefit welfare as well as performance [52]. Public outreach has been suggested as a method to apply scientific discovery to horse training, breaking down barriers and challenging long-held beliefs [51]. However, depending on individual beliefs, equestrians can be sceptical about science [53]. Without sensitivity to this, suggesting such solutions would only continue the apparent mismatch between theoretical knowledge and field applications [11]. While scientific advances can be communicated simply, there is currently no evidence to suggest that solely increasing awareness improves welfare assessment skills, although this may be a limitation of the study design rather than evidence of no effect [13]. Similarly, for management practices, a recent study delivering research-based information to horse owners via webinar found an increase in knowledge but was not able to assess whether the information was applied or skills increased [53]. Therefore, further research may be required to assess the impact of increased knowledge or awareness on horse welfare in practice. For example, research that analyses the delivery of welfare education could include or lead to a follow-up study that assesses the welfare of horses under the care of attendees and evidence of any changes in their management after the learning experience to provide evidence for or against this as a welfare improvement strategy.

### 4.2. Recent Developments in France

In France, the horse population has been expanding since 1995, reaching 950,000 in 2010 [54]. This growth is the result of the development of pony riding for children and the increasing interest of French people in recreational riding and horse betting [55]. Concerns in France are like those in the UK: human–horse relationships, economic efficiency, environmental issues, preservation of horse breeds, land-use pressures, and the health, welfare, and care of animals, including death [54].

In 2021, France introduced new legislation, to be brought into effect in 2022, that would require all horse owners to undertake education to demonstrate their knowledge about equine management [56]. This education strategy is different from the public outreach to raise awareness discussed above, as it has a more formal course and teaching structure with evidence of knowledge required. The goal of this legislation and education programme is to combat abuse, and the initiative has been lobbied for since the introduction of licensing for Federation Française D’Equitation (FFE) members in 2019 [16]. This presents an interesting idea for other countries experiencing persistent cases of compromised equine welfare. The French initiative would appear to target the leisure riding community, as competitive riders are already required to have a membership [57]. To be licensed, owners and riders have to complete the Gallop 4 qualification, or an equivalent, that covers general horse knowledge, horse care, equestrian practice and safety practices.

With the growing understanding of human behavioural science [58] in mind, consideration of horse-owner values and perceptions should inform the practicality and viability of applying a similar change in legislation in the UK. When applying human behaviour change principles to a specific horse welfare concern, obesity, it was found that environments and social norms limited the likelihood of proactive action by horse owners, and there were issues with owners’ ability to identify and assess such health issues or weigh up the short-term impacts of weight management against the long-term health benefits [59]. Although that study was focused on one specific area of horse welfare, it covered a significant sample size and effectively applied a mixed methods approach to obtain a holistic view of the issue; therefore, this method could be extrapolated to consider other welfare issues in turn. However, the questions asked in this study remain: can education, either voluntary or mandated through a licensing system, overcome barriers to human behaviour change and directly benefit animal welfare?

### 4.3. Education and Licensing in Principle

While this study aimed to explore both education and licensing with respect to their ability to improve horse welfare, less research was conducted on the successes and limitations of licensing than on education. Thus, there is a skew in the discussion and analysis towards the costs and benefits of education and barriers to this as a solution to horse welfare concerns. As education appears to be the primary objective of the French horse-owner license, and the results suggest that successful licensing relies upon education (Figure 8), it is reasonable that this discussion places a greater focus on education. However, licensing will be explored, with the potential for combined enforcement and independent regulation.

#### 4.3.1. Education and Horse Welfare

Education has the potential to make a difference in horses’ welfare through their owners; in fact, educating owners has been argued as combatting poor welfare in other domestic species [60,61]. The lack of knowledge is the highest-cited barrier to horse welfare (e.g., [42]), with a lack of understanding of the available information about horses’ needs being second (e.g., [62]) (Figure 3). Concurrently, increased knowledge, awareness and understanding were the most successful aspects of educational interventions (e.g., [63]) (Figure 5), with increased awareness being the second-ranked solution to poor horse welfare (e.g., [64]) while education was the fourth (e.g., [65]) (Figure 4); however, barriers to increasing knowledge and understanding are similar to those discussed above. Cultural change was the second-ranked education success (e.g., [66]) (Figure 5); however, the most common education limitation is an unclear effect on human behaviour, (e.g., [59]) (Figure 6). Cultural change can be shown in non-material forms (e.g., language), and this may explain its high ranking in education success; however, material changes (e.g., practical application) are needed for modifications that affect horse welfare. Therefore, this calls into question the usefulness of education in creating material culture/behaviour changes (third-ranked solution) as a method to improve horse welfare (e.g., [67]) (Figure 4).

To explore this further, we must look in more detail at other factors involved in horse welfare. Notably, amongst the causes of welfare concerns are the following: competition, attitudes/beliefs and owner perception, as well as limitations of facilities (Figure 2). The first three of these are within the owner’s control: what goals they set, what they believe is required or right to achieve their goals, and what they see as good or bad for welfare and performance. While education may influence the culture around these facets of horse welfare (Figure 5), the previously highlighted discrepancy calls into question whether it will lead to meaningful human behaviour change (Figure 6). Limited facilities are an interesting addition to the data set, as these could be beyond an owner’s control, such as within the context of a livery yard. Livery restrictions have been suggested as a barrier to lasting behavioural change following educational intervention [13]. Facility limitations have been associated with cited welfare concerns, such as behavioural problems [68], weight problems [44], confinement and isolation [49] or crowding (insufficient space) [69]. Overpopulation, cited as another cause of poor welfare (e.g., [41,68]), could exacerbate limited facilities; if there are too few well-equipped facilities for the horse and owner population, then owners’ choices become limited. Overall, facility limitations may also limit the success of an educational remedy to compromised horse welfare, unless other regulations are introduced [70]. Another challenge is that privately owned horses may be kept in a variety of environments aside from livery yards, such as the owner’s home property. Facility limitations of these environments may still exist but may also be different than those of horses housed in livery. Exploring the differences in welfare concerns and the potential opportunities and challenges of education and licensing to improve welfare between different populations of privately owned horses was out of the scope of this review but may provide interesting data in future research that could help tailor recommendations to specific populations.

The issue becomes more complex when considering that beliefs, resistance, lack of trust, and personal bias are also potential barriers to good welfare (e.g., [8]) (Figure 3). Additionally, financial constraints, circumstances, awareness, communications, and lack of clarity all may influence the ability of education to improve individual horse welfare [58]. The last three of these factors are also affected by the horse industry and the government, who have the ability to provide clear communications and ensure that prominent issues are transparently presented with any disparities between advice streams being resolved [70]. This would follow suggestions two and five of solutions to welfare concerns (Figure 4—increased communications and industry-led initiatives/commitment). However, wider education resources may be needed to ensure an understanding of complex issues by novice owners, as well as the uptake of new ideas [71]. The first three barriers mentioned above (beliefs, resistance/lack of trust, and personal bias) are also complicated as they are rooted in an individual’s culture, experience and previous education [51]. Each individual has their own perception of what good horse keeping entails [72]. This is where communications can still play a key role; using appropriate terminology and communication channels, trusted sources and trying to align messages within existing belief frameworks make it more likely that important messages will be positively received by horse owners [73]. Trusted sources are often known contacts or groups [45]; however, there may be scope for experts on specific welfare topics to raise awareness within these smaller communities and build trust with private horse owners [74]. On the other hand, financial constraints and other circumstances are challenging barriers to overcome, and it is significant that wider circumstances are highly ranked as a limitation of education (Figure 6). These restrictions are influenced, in turn, by a wide range of other factors, including the economy, work pressures, geographical location, life changes, and other individuals’ actions [61]. These circumstances can make it hard for theory to be put into practice; an owner can know the optimal way to care for their horse but hit a roadblock if they do not have enough time, or if the cost of essential horse maintenance increases. This barrier would require more than well-designed communications to be overcome, indicating that education can target some of the root challenges to good horse welfare; however, it cannot reduce non-horse-related external pressures on horse owners.

We should also look closely at other limitations of education. The unclear effect on human behaviour is the most problematic, as an overall change in attitude and behaviour towards more welfare-friendly practices is evidently sought after throughout the literature (Figure 4) [75]. In 2012, Visser and Van Wijk-Jansen [45] found that many participants had good awareness of issues compromising horse welfare; however, their knowledge was not consistent in producing appropriate practices. There could be many reasons for theory not translating into application by horse owners, including external factors. A particular barrier that has been suggested is the perception of the issues, which ranks fourth as an education limitation (Figure 6) (e.g., [76]). Thompson and Clarkson [73] suggested that there was a disparity between owner self-reporting and observations by a trained professional regarding horse behaviour. Furtado et al. [77] also cited that owners demonstrated inaccuracy in assessing their horse’s weight. The perception and prioritisation of issues are linked closely to personal beliefs and biases that may be barriers to welfare (Figure 3) [9]. There is scope for education to reduce this barrier, as education has been found to improve openness to new ideas (Figure 5) [78]. However, there is some ambiguity around this, which will be explored further.

Availability and syllabus content are ranked as the second and third limitations for education to be successful (Figure 6). Availability can be affected by other limitations, including resource (e.g., [78]) and resistance, alongside lack of uptake (e.g., [28]) (Figure 6). Making education available is challenging as it requires infrastructure; institutional/national support is the highest-ranked input for education to be successful (Figure 5) (e.g., [79,80]). Justification of resourcing is difficult if there is a lack of uptake, and stakeholders have expressed scepticism about whether those who really need education would be likely to volunteer for learning or be truly impacted by the information [27]. Although this belief may be biased within the small sample of the Collins et al. study [27], the concern would appear to be supported by the findings here, as education’s limitations include resistance and limited influence (Figure 7), as well as a limited effect on behaviour as discussed above. Uptake becomes less of a problem if education is made mandatory. The question is then about the likely effectiveness and the value of implementation. On the other hand, syllabus content can be controlled with sufficient collaboration between governing bodies, key stakeholders and education providers. A modern syllabus that is up to date with recent scientific findings and the range of currently held beliefs in the community could improve the appeal of voluntary education. The problem, then, is agreement over delivery. There is a lack of consensus in the literature over how best to deliver welfare education, with arguments being made for interactive [79,81], online [78,82], communication-based [83], animal-centred [84], multilevel [85], interdisciplinary [86,87] and assessed methods [84]. The idea of online learning also has merit. MacKay et al. [78] found that Massive Open Online Courses had a high success and acceptance rate throughout a large and diverse sample of participants. The COVID-19 pandemic also created an increase in testing and delivering online learning. Sasaki [82] found that practical horse training for veterinarians could be delivered online without compromising achievement, maintaining student satisfaction and increasing self-directed learning. While this was a pilot study, it had a straightforward and repeatable method that could be worth expanding for generalisability. The sample in the Sasaki study [82] was also well balanced in terms of participant gender, which is unusual amongst the assessed literature, which tends to be majority female, indicating a high potential accessibility or appeal of online learning. Further research or consultation would be required to ascertain which method of delivery would be optimal for horse welfare education.

There is a discrepancy in the results as to whether education improves individuals’ ability to respond to change. One of the goals for improving welfare is to move away from outdated ideas [10]. Ten papers cite being open to new ideas as an education success (Figure 5) (e.g., [88,89]); however, four papers cite reduced perspective taking as a limitation (Figure 6) (e.g., [90]). To determine whether education is a useful tool towards this goal, it needs to be discerned whether education is likely to enable horse owners to change their practices or if it may limit their perspective of the issues. As all the respective papers within the assessed literature are mainly focussed on meat production and consumer choice, further research is needed to determine specifically whether education can influence horse owners’ reception of new information.

There is also a question around improved skills, in this case, the ability to improve horse welfare through management and training practices. Studies have found correlations between human skills and animal welfare [90,91]. Improved skill is ranked as an education success by four papers in the assessed literature, (e.g., [91,92,93]) (Figure 5); however, one paper cited a lack of improved skill following education (Figure 6) [12]. The study by Fletcher et al. [12] did not find a difference in participants’ skills to detect welfare parameters based on their level of equine-related academic qualifications. This could be indicative of a lack of focus in the syllabus on welfare assessment or a lack of distinction in the syllabus regarding welfare definitions. It should also be noted that the sample size for that study was small compared to the representative population and would need expanding to truly evaluate any potential differences in educated and non-educated equestrians’ abilities. Again, the papers advocating for education to increase skill levels tend to be focussed on the production of animals [91,92] and improving young adult nutrition [94]; thus, their applications may require testing in the horse-owning community. Further research is needed to assess whether basic education can improve the skills of horse owners with regard to improving welfare standards.

#### 4.3.2. Licensing for Regulation

Regulation is discussed in the literature as a possible solution for horse welfare issues but ranks very low in the suggested solutions (Figure 4). This could be due to a lack of consensus amongst stakeholders about what to regulate and how [27] as well as the cost and resources required, such as time and training of regulators, to implement such a programme [40]. However, it should be noted that licensing provides regulatory opportunities. Regulation is the second-highest-ranked licensing success in the literature (Figure 7). A recent study by Liang et al. [95] suggests a need for a security system for animal welfare, using licensing as a regulatory tool. That study was focussed on consumer choice regarding meat consumption; therefore, care should be taken when considering the findings with regard to horse welfare. Also, the concern for security may have been influenced by the COVID-19 pandemic; thus, the experiment would benefit from fresh application to the privately owned horse community. However, increased levels of regulation, potentially through tighter licensing, have also been suggested to safeguard wild animal welfare in the UK [96], suggesting potential for regulation to be promoted across the wider animal welfare sector.

Additionally, increased reporting has been advocated for with regard to horse welfare. A lack of monitoring and reporting is listed as a significant barrier to good horse welfare (Figure 3), and improved reporting is suggested as a solution to horse welfare issues (Figure 4). At the same time, this is identified as a licensing success (Figure 7). A lack of reporting is noted as a problem for determining the welfare status of a population [44], a threat to welfare itself [97] and assessing welfare concerns in sporting scenarios [98]. The database study by Hitchens et al. in 2017 [99] made a compelling case for using an inspection-informed database to monitor animal welfare regionally and internationally. However, this study was reliant on modelling and markers to analyse the data due to the size of the data set, and there was also a risk of inspector bias as they may have been influenced by employer or individual agendas. The paper also notes that some desirable data were lacking in the database, making this method of assessing prevalence currently limited, although promising. The measures of equine welfare in this study do not cover all aspects of welfare; thus, the efficacy of a comprehensive inspection reporting system still needs to be tested. For a reporting system such as this to be implemented, the challenges of cost and centralisation still need to be overcome [97], and then the discussion circles back to how best to assess welfare [44]. However, if it can be implemented correctly, the literature overall appears to support the use of licensing as a reporting tool for the benefit of horse welfare.

A notable challenge when licensing, for any purpose, is ensuring the system works. The most-cited licensing limitation is enforcement (e.g., [27]), followed relatively closely by resources (e.g., [41]) (Figure 8). There are also existing concerns within the literature about the effectiveness of legal systems to enforce current legislation regarding horse welfare [72,100] as well as animal welfare more generally [101]. If resources for animal welfare regulation are already stretched, it could be unreasonable to add to this burden until wider socio-economic issues are resolved. On the other hand, a reduction of animal welfare cases requiring charitable or legal intervention would reduce the cost placed on protecting animal welfare. Thus, it may need to be considered whether short-term investment is necessary for long-term alleviation. There are also concerns that licensing only enforces minimum standards rather than promoting high welfare [102,103], which can, in turn, lead to a lack of trust by the public regarding whether the license is meaningful [100,102]. The considerations for introducing a licensing system in the UK will be explored further below.

#### 4.3.3. Licensing for Education

Being supported by education is listed as one of the top three factors for licensing success (Figure 7). However, there is nothing in the assessed literature to suggest that licensing aids education success. This calls into question the applied usefulness of licensing with regard to horse welfare when used in tandem with education.

Despite this, there are parallels between licensing and education successes and limitations in the assessed literature. National support is highly cited as a required factor for both education, (e.g., [69]) and licensing, (e.g., [104]) to be successful (Figure 5 and Figure 7). Cultural change is also the second-highest cited education success (Figure 5) and ranks in the middle for licensing successes (Figure 7) (e.g., [105]). Additionally, despite some papers advocating that licensing can promote human behaviour change [106], the overall results list a limited influence on human behaviour as a licensing limitation (Figure 8), as with education (Figure 6). It should be noted that the study by Tickle et al. [107], which showed a positive influence on human behaviour from licensing, focussed on a niche group with regard to welfare and licensing, specifically young people involved in hunting in Sweden; thus, the wider literature is more likely to be indicative of the ability of licensing to incite behaviour change as a general principle.

Resource is also a limitation of both education and licensing (Figure 6 and Figure 8). Introducing new licensing requirements dependent on a new education system would be resource heavy and this could prove to be a major barrier to introduction in the UK. By contrast, in France, the licensing and education framework already existed for the competitive riding community [57]; therefore, the new legislation required minimal additional infrastructure or resources. If such a system were to be implemented in the UK, it is possible that a similar gradual approach would be required. Uptake of licensing, or lack thereof, is another highly cited limitation (Figure 8), which again rings true with the previously discussed education limitations. Most studies looking at voluntary licensing schemes are focussed on production animals, (e.g., [93,108]); however, similar problems were found by Collins et al. [97] when exploring horse welfare issues in Ireland. To overcome this, the promotion and introduction of a licensing and/or education system would need to be aligned with the motivations of horse owners in the UK. Finally, it is important to note that a lack of clarity and transparency are both listed as licensing limitations in the literature (Figure 8). A key factor in licensing success would be to make it clear what the license means and what it aims to achieve [109]. In France, the legislation has been stated as combatting horse abuse [16]. However, if individuals who mistreat horses understand it is wrong, as suggested by Collins et al. [100], then, in practice, licensing that targets other welfare issues that do not stem from ignorance or the licensing is being brought in for additional reasons, such as monitoring. Depending on the culture around bureaucracy in each country, public acceptance of a new initiative may be vastly impacted by the openness with which the aims of that initiative are communicated. The considerations for licensing to be accepted in UK society are discussed further later.

### 4.4. Further Considerations for Education

When exploring the possible application of education, thought should be given to how this can be perceived by separate groups within the target population. A key finding from this study was that education needs to be available (Figure 6), which indicates it needs to be delivered in an accessible format and marketed appropriately. Although there have been suggestions to incorporate basic welfare education into the school curriculum, which has been piloted [61,76,80,110], this study is focussed on horse-owner (adult) education; therefore, the applications of this are not discussed in detail.

As horse-owner education is primarily for adults, related education research should be considered. Adults typically take an interest in learning that has immediate relevance to their lives [111]. Knowles’ theory of Adult Learning suggests that learning needs to be collaborative [112], which supports arguments for any form of education for horse owners to have a discursive, communicative element. Additionally, adult learning is self-directed, problem-based and focussed on self-directed learning goals [113]. Adults generally seek education for reasons they perceive as meaningful and will determine the level of responsibility they wish to take [114]. Thus, relating welfare education to horse-owner motivations is a priority, especially to involve those who have the least interest but possibly the greatest need for learning [27].

Gender should not be ignored when considering the findings of this study, as the vast majority of assessed papers involving horse owners have a skew towards women participants. This could be a self-selecting sampling bias or incidental due to recruitment channels. Regardless of the reason, the gender skew in the current research pool has implications for drawing conclusions and next steps. Research has shown that differences still exist between male and female psychology [114]. Notably, Fiedler et al. [115] found that males were less likely to listen to and act on welfare messages. Barriers to education can also be influenced by gender. Women are more concerned about missing work for learning, which may stem from less stable or flexible working arrangements [116]. Women are also more likely to be affected by barriers to education, such as financial constraints and spending time away from family [116], and may be apprehensive of online learning due to ease of use and computer self-efficacy [117]. This suggests that while women may be more open to welfare messages, they feel less able to take on additional learning. Thus, education for horse welfare based on existing research should be designed appropriately; for example, e-learning environments can be more effective for women when they allow for peer-to-peer communication and connectedness [118]. Differences in motivators for education have been also found between genders. For example, females draw motivation from a supportive network of individuals [116]. Additionally, 82.8% of surveyed horse enthusiasts (mostly female) identified other enthusiasts, arguably a close network, as their most important source of information [46]. Although a more recent, smaller survey by Pickering and Hockenhull [74] found that 18.7% of horse owners cited veterinarians as the information source most likely to be consulted compared to 13.1% prioritising other horse owners, the design of that survey asked respondents to make choices in given scenarios and not all participants selected the maximum number of choices available. By contrast, Visser and Wan Wijk-Jansen [46] used open questions about information-seeking behaviour. Thus, the results of the later study do not necessarily indicate general day-to-day information-seeking preferences of horse owners. Therefore, a successful method to motivate female horse owners to learn more about horse welfare could be through popular information sources already feeding into trusted networks. By contrast, if an education initiative is to avoid the risk of excluding men, further research is needed to ensure their views are fully understood regarding barriers to horse welfare.

Horse-owner education should be applied to cater to adult learning styles [113]. This is not necessarily a guaranteed success for welfare education, as some research has found that some pre-existing beliefs may persist if they are rooted in deeply held values [119]; however, the evidence indicates it would increase the likelihood of success. Delivery also needs to be within the national legal framework. In the UK, policy regarding education can vary, sometimes suddenly and with rapid change or reversal, depending on the government in power [120]. Thus, all adult education is voluntary unless required for a particular reason, such as a job role [121,122]. However, a general premise for licensing to mandate education does exist as adults are assessed to be licensed to drive [122]. Therefore, there is scope to introduce a similar system within the UK’s legal framework. However, the present UK policy prioritises learning for young children and upskilling for economic growth [123], meaning that animal welfare education programmes for adults may require significant lobbying to become a high-level concern.

### 4.5. Further Considerations for Licensing

When exploring possibilities for improving monitoring and reporting through licensing, thought should also be given to the national feeling. The UK has been suggested as a low power distance culture, responding positively to public voice opportunities for new projects that directly affect individuals [124]. Other countries with high power distance cultures can be more favourable to new projects decided and implemented without consultation [125]. This suggests that a new licensing system in the UK would be better accepted following public consultation. Interestingly, the survey by Visser and Van Wijk-Jansen [46]—conducted in the Netherlands, another low power distance culture [125]—found that 55.1% of respondents were in favour of governmental regulation for horse welfare. This corresponds with findings by Collins et al. [27] that equine stakeholders believe regulation to be the most effective means for raising welfare standards. However, there remains ambiguity in the existing research about what form this regulation should take.

Furthermore, there could still be resistance to any proposed change regarding licensing due to the culture of bureaucracy in the UK. Whereas countries such as Germany still follow a hierarchical organisation and can be quite accepting of regulation, the UK’s values are based on a more pragmatic approach, where bureaucracy is less present and there is a shift towards more commercial values [126]. While hierarchical bureaucratic systems have their problems, so do their alternatives. Managing through empowerment risks generating outcomes that were meant to be avoided, including de-professionalisation, removal of support networks and the lack of a conflict resolution framework [127]. It could be argued that this has been the approach to horse welfare in the UK. The Animal Welfare Act [128] places responsibility on owners/caregivers to understand and follow the law with regard to the horse’s needs [35], ownership is not governed, regulation of horse registration can be inconsistent [27], and welfare concerns are generally responded to by charitable bodies like the Royal Society for the Prevention of Cruelty to Animals, which often rely on public donations. Perhaps increased bureaucratic intervention is needed to resolve horse welfare concerns in the long term. However, as with education, a regulation system will require resources, and these may not be justified if the system cannot work due to resistance in the public. Bureaucracies need to be impartial as well as efficient for citizen satisfaction [129]; therefore, a new bureaucratic system should be objectively justified to the public to increase the likelihood of effectiveness.

While this study has distinguished between licensing specifically based on educational requirements and licensing for other purposes, these are not mutually exclusive concepts. In fact, Liang et al. [95] suggested providing animal welfare education as well as security systems for animal welfare. This is not the only study to highlight a need for multi-level intervention for animal welfare. May and Previte [66] propose treating animal welfare issues, such as overpopulation, as a network across which social communications (marketing) and education combine to achieve effective regulation. Furtado et al. [77] also propose a holistic approach to achieving human behaviour change, and Douglas et al. [67] argue that the approach to protecting horse welfare needs to be holistic to protect the industry’s social license to operate. Even private horse owners may be affected by a social license to operate, as they are subject to public exposure and could be viewed as representatives of the horse industry as a whole. This approach to welfare marries up with suggestions in the literature that sources of welfare concerns, such as management systems, need to be assessed as a whole, not by parameter [45]. Holistic change also creates the opportunity to apply a collection of strategies with a view to tackling different welfare concerns according to their particular causes. However, one overarching policy and industry change contradicts suggestions to prioritise resources on key welfare issues in the UK [18,42]. The answer to this riddle could be that it is impossible to please all stakeholders, which should not be surprising given the disparities in views explored in Section 4.1. As an alternative, an initial holistic policy change could be implemented, which is then followed by targeted interventions for prioritised welfare concerns to allow for gradual changes to improve horse welfare that may be more practical to implement.

### 4.6. General Discussion

This study has demonstrated the need for sector-wide changes to improve horse welfare. This concurs with a recent report by the RSPCA citing regulation, human behaviour change and increased knowledge as primary focuses for tackling the equine crisis [130]. Overall, the findings of this study indicate that education could increase horse-owner awareness of welfare issues; however, there is a lack of evidence to suggest that licensing directly enhances horse-owner knowledge or ability. It appears that education can improve individual commitment to welfare and acceptance of current ideas while being a popular solution to horse welfare issues. However, to be effective, information sources need to be harmonised, education opportunities need to be accessible to a diverse audience and communication channels optimised for the characteristics of the horse-owning community. However, education also seems to have a limited influence over human behaviour change; thus, it is questionable whether education alone is likely to reduce cases of compromised horse welfare in the UK. Additionally, literature is divided over whether actions to improve horse welfare should prioritise and target the most prominent concerns or apply a holistic blanket approach. Neither method applied in isolation is likely to succeed in resolving all horse welfare concerns; therefore, there is a case for prioritising resources on interventions likely to succeed in combatting the most serious infractions. Licensing can potentially induce cultural change, improve monitoring and aid consumer decision making. However, licensing is most limited by available resources and cost, and there is limited evidence in the literature that licensing can improve welfare standards within a population, although it could aid monitoring and regulation and support or be supported by education. This indicates that licensing horse ownership without education is unlikely to positively impact the welfare state of the UK horse population. However, there is more limited data available for licensing with regard to animal welfare; therefore, more research is needed in this area. Finally, although mandating education and regulating horse ownership has merit in improving the knowledge level within the community, care is needed regarding the bureaucratic culture in the UK compared to other countries. Additionally, education and licensing can both be limited when applied to combat specific welfare concerns, yet it is doubtful that one solution can achieve a blanket improvement in horse welfare in the UK; therefore, other systems of regulation and support may be required.

This study only discussed the most prevalent horse welfare concerns found, as issues with minimal data behind them would be limited in their generalisability to the wider horse population. Additionally, exclusion criteria may have led to some valid yet not-cited papers being overlooked, or health issues mainly studied in commercially owned horses (e.g., racehorses or brood mares) being underrepresented. However, excluded papers that appeared in the database searches were still briefly reviewed, meaning the potential for an important paper to be excluded from this study was minimised. The lack of peer-reviewed publications around horse welfare and licensing, leading to the inclusion of any animal welfare licensing, does highlight the need for this research to be conducted if the use of licensing requirements is to be promoted as part of a solution to horse welfare concerns.

Overall, this study is predominantly theoretical. The next steps for this research would be to investigate the successes and limitations of the newly introduced education and licensing framework in France. In particular, the number of owners previously without a license becoming registered over time, and the number of horse welfare concerns reported in the same time frame compared to before the new legislation. Additionally, data should be collected on horse-owner perception of the new initiative and their knowledge of welfare issues. That data could then be compared with the findings of this study to test the accuracy of these theoretical conclusions and inform future work in this area in relation to UK privately owned horse welfare.

## 5. Conclusions

In conclusion, there are mixed results as to the potential success of using education and licensing to improve privately owned horse welfare in the UK. However, some aspects look promising, such as increased awareness of horse welfare issues with the combination of an education and licensing scheme seeming to have some potential for success. Due to the complex nature of horse welfare issues, educational inventions and licensing systems, careful consideration of format and application needs to be taken before implementation. Further research into these issues would improve chances for success and future improvements in horse welfare.

## Figures and Tables

**Table 1:** Search terms used.

Search Engine	Search 1	Search 2
Web of Science	Horse* OR Equine* (Title) AND welfare OR wellbeing OR wellbeing (title) AND “animal welfare” (Topic) OR Horse* OR Equine* (Abstract) AND welfare OR wellbeing OR wellbeing (Abstract) AND “animal welfare” (Topic)	Education* OR Licensing* (Title) AND “animal welfare” (Topic) OR Education* OR Licensing* (Abstract) AND “animal welfare” (Topic)
Google Scholar	Allintitle: “horse welfare”/“horse wellbeing”/“equine welfare”/“equine wellbeing”	Allintitle: “education animal welfare”/“licensing animal welfare”

**Table 2:** Frequency and spread of inputs by theme.

Theme	Input Frequency	Input Variation
1—Welfare Concerns	248	17
2—Causes of Poor Welfare	197	17
3—Barriers to Good Welfare	168	18
4—Solutions	177	13
5—Education Successes	167	21
6—Education Limitations	119	18
7—Licensing Successes	48	15
8—Licensing Limitations	65	19

**Table 3:** Most cited inputs per theme (modal theme).

Theme	Highest Input Frequency
1—Welfare Concerns	Distress/stress/fear
2—Causes of Poor Welfare	Management practices
3—Barriers to Good Welfare	Knowledge
4—Solutions to Welfare Issues	Further research
5—Education Successes	Increased knowledge/awareness/ understanding
6—Education Limitations	Unclear effect on behaviour
7—Licensing Successes	Consumer tool
8—Licensing Limitations	Enforcement

## Data Availability

A full list of included papers is available in Appendix B. A list of excluded papers and the full summarized data are available from the author if requested.

## Appendix A

**Table A1:** Completed PRISMA protocol for the completion of data collection.

Section/Topic	#	Checklist Item	Information Reported		Line Number(s)
			Yes	No	
Administrative information					
Title					
Identification	1a	Identify the report as a protocol of a systematic review.			111
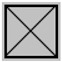
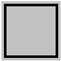
Update	1b	If the protocol is for an update of a previous systematic review, identify as such.			
Registration	2	If registered, provide the name of the registry (e.g., PROSPERO) and registration number in the Abstract.			
Authors					
Contact	3a	Provide name, institutional affiliation, and e-mail address of all protocol authors; provide physical mailing address of corresponding author.			5, 6
Contributions	3b	Describe contributions of protocol authors and identify the guarantor of the review.			816–822
Amendments	4	If the protocol represents an amendment of a previously completed or published protocol, identify as such and list changes; otherwise, state plan for documenting important protocol amendments.			
Support					
Sources	5a	Indicate sources of financial or other support for the review.			
Sponsor	5b	Provide name for the review funder and/or sponsor.			
Role of sponsor/funder	5c	Describe roles of funder(s), sponsor(s), and/or institution(s), if any, in developing the protocol.			828
Introduction					
Rationale	6	Describe the rationale for the review in the context of what is already known.			42–97
Objectives	7	Provide an explicit statement of the question(s) the review will address with reference to participants, interventions, comparators, and outcomes (PICO).			98–108
Methods					
Eligibility criteria	8	Specify the study characteristics (e.g., PICO, study design, setting, time frame) and report characteristics (e.g., years considered, language, publication status) to be used as criteria for eligibility for the review.			110–178
Information sources	9	Describe all intended information sources (e.g., electronic databases, contact with study authors, trial registers, or other grey literature sources) with planned dates of coverage.			110–178
Search strategy	10	Present draft of search strategy to be used for at least one electronic database, including planned limits, such that it could be repeated.			110–178
Study records					
Data management	11a	Describe the mechanism(s) that will be used to manage records and data throughout the review.			
Selection process	11b	State the process that will be used for selecting studies (e.g., two independent reviewers) through each phase of the review (i.e., screening, eligibility, and inclusion in meta-analysis).			124–151
Data collection process	11c	Describe planned method of extracting data from reports (e.g., piloting forms, executed independently, in duplicate), any processes for obtaining and confirming data from investigators.			153–171
Data items	12	List and define all variables for which data will be sought (e.g., PICO items, funding sources), any pre-planned data assumptions and simplifications.			153–171
Outcomes and prioritization	13	List and define all outcomes for which data will be sought, including prioritization of main and additional outcomes, with rationale.			153–171
Risk of bias in individual studies	14	Describe anticipated methods for assessing risk of bias of individual studies, including whether this will be performed at the outcome or study level, or both; state how this information will be used in data synthesis.			
Data					
Synthesis	15	Describe criteria under which study data will be quantitatively synthesised.			153–171
	15b	If data are appropriate for quantitative synthesis, describe planned summary measures, methods of handling data, and methods of combining data from studies, including any planned exploration of consistency (e.g., *I*^2^, Kendall’s tau).			153–171
	15c	Describe any proposed additional analyses (e.g., sensitivity or subgroup analyses, meta-regression).			
	15d	If quantitative synthesis is not appropriate, describe the type of summary planned.			
Meta-bias(es)	16	Specify any planned assessment of meta-bias(es) (e.g., publication bias across studies, selective reporting within studies).			
Confidence in cumulative evidence	17	Describe how the strength of the body of evidence will be assessed (e.g., GRADE).			

## Appendix B

This appendix provides definitions to describe how each search was broken down into themes and corresponding inputs. Table A2 defines each of the eight themes, Table A3 and Table A4 define each input in the order they are ranked and, for Search 2/Themes 5–8, identifies whether they are contributory or outcome factors.

**Table A2:** Definitions of Themes.

Theme	Search	Definition
1—Welfare Concerns	1	Horse welfare concerns highlighted by the literature
2—Causes of Poor Welfare	1	Factors identified by the literature as increasing the risk of horse welfare concerns or directly causing them
3—Barriers to Good Welfare	1	Factors identified by the literature as making good welfare more difficult to achieve or preventing changes to improve horse welfare
4—Solutions to Welfare Issues	1	Suggestions in the literature to improve horse welfare
5—Education Successes	2	Factors identified in the literature as increasing the likelihood of education being effective (contributors) plus positive outcomes of education (outcomes)
6—Education Limitations	2	Factors identified in the literature as decreasing the likelihood of education being effective (contributors) plus negative or lacking outcomes of education (outcomes)
7—Licensing Successes	2	Factors identified in the literature as increasing the likelihood of licensing being effective (contributors) plus positive outcomes of licensing (outcomes)
8—Licensing Limitations	2	Factors identified in the literature as decreasing the likelihood of licensing being effective (contributors) plus negative or lacking outcomes of licensing (outcomes)

**Table A3:** Definitions of Inputs from Search 1.

Input	Theme	Definition
Stress/distress/fear	1	This includes any references to horses experiencing undue/avoidable stress or fear or being in distress.
Health	1	This includes all health related welfare concerns excluding explicit references to pain, lameness, obesity and other concerns that were sufficiently referenced to have their own category.
Conflict/stereotypic/hyperactive behaviours	1	This refers to a variety of unwanted behaviours that have been recognised as welfare concerns. Conflict behaviours include those interactions that are both between horses and other horses and horses and people. Stereotypic behaviours include weaving, box walking, crib biting and wind sucking.
Confinement	1	The prolonged restriction of movement and space, often through long-term stabling.
Pain	1	Includes cited concerns over horses experiencing pain as a direct result of human interaction or care.
Isolation	1	The limitation or prohibition of socialisation with other horses, for example, through prolonged stabling or mandated individual turnout.
Injury	1	Specific reference in the literature to injury as a welfare concern, for example, training or competition induced or as a result of poorly maintained facilities.
Coercive control/equipment (e.g., restrictive tack, bits)	1	Cited concerns about the use of restrictive equipment or practices that aim to apply control and/or force to achieve training aims.
Obesity	1	The specific concern about horses being overweight and the impact of this on their welfare.
QoL	1	Quality of Life. Specifically cited references to reduced QoL as a welfare concern.
Affective State (inc. confusion and withdrawal)	1	References to negative affective states as a welfare concern, including learned helplessness, but not the previously mentioned unwanted behaviours. This is distinct from the behaviour category, as these states can be a cause or effect of such behaviours or completely unrelated, i.e., a horse may present with no clear unwanted behaviours but be experiencing a negative affective state.
Cruelty/abuse/neglect	1	Direct action or inaction of human caregivers as a welfare concern. Although these can also be causes of welfare concerns, the literature refers to them as concerns in their own right on a number of occasions.
Work (inc therapeutic activities)	1	Concerns directly resulting from or relating to the use of horses in a working (non-sporting) capacity, for example carriage horses, therapy horses, etc.
Crowding	1	The overpopulation of horses in a given space, for example, an over-full livery yard without sufficient turnout or stables for horses to receive optimal management and/or nutrition.
Lameness/hoof care	1	Concerns specifically cited in relation to horses being unsound and poor hoof maintenance.
Emaciation	1	The concern about horses being severely underweight.
Sleep deprivation	1	The concern about horses not being able to get enough proper sleep and the resulting consequences.
Abandonement	1	The concern about horses being simply left by their owners; this may be extreme, such as being left by a roadside, or more complex scenarios whereby owners leave their horse in someone’s care and disappear or stop paying for this service.
Management	2	All potential causes of poor horse welfare that relate to how the horse is kept, excluding feeding, as this was referred to frequently enough to warrant its own category. This also includes “standard” management techniques that have fallen under criticism.
Training	2	All potential causes of poor horse welfare relating to equitation in any discipline, excluding tack use, as this warranted its own category. This includes both in-hand and ridden training.
Tack (inc. bit use)	2	References to tack as a cause of poor horse welfare, with a special note regarding RE bit use, as this was a frequently explored example.
HAI (inc. veterinary interactions etc.)	2	Human–Animal Interactions. This category includes references to how the interaction with people (caregivers and less familiar persons) can compromise horse welfare.
Competition/performance gain	2	Citations listing the desire to win or improve as a potential cause of reduced horse welfare, including the prioritisation of such goals as well as the impact of practices employed to achieve them.
Limitations of facilities	2	Specific references to available stables/turnout/training spaces as causes of poor welfare when restricted.
Attitudes/priorities/moral beliefs	2	A collective category for cited instances where people’s individual or group norms are a cause of compromised horse welfare
Ineffective regulation/legislation	2	References to policy and enforcement, or a lack thereof, being a cause of poor horse welfare.
Feeding Regimes	2	Specific references to feeding practices being a cause of poor welfare.
Owner perception	2	Incidences where horses’ caregivers’ views or understanding of an issue can lead to a welfare concern.
Social Norms	2	Identified citations that list commonly held beliefs as a cause of compromised welfare.
Rider/owner skill (inc. posture/balance)	2	The individual ability of people responsible for horse training or management as a cause of welfare concerns, including their physical abilities and their level of skill in assessing the potentially changing needs of a horse.
Rider/owner psychology	2	The impact of individual mental wellbeing and processing on horse welfare.
Overpopulation	2	This includes localised issues of having too many horses in a space and the overarching issue of there simply being too many horses for the number of responsible owners available, both of which can compromise horse welfare.
Retirement	2	The issue of horses being retired from work and incidences where, when carried out poorly, this has compromised horse welfare.
Lack of training (people)	2	References to situations where horse welfare has been impaired by those responsible not having sufficient training.
Complexity of issue	2	This input is for instances where the complexity of horse welfare itself has been identified as a cause of poor welfare, i.e., there are a huge number of interrelated factors that can be hard to assess and balance especially in performance horses.
Knowledge	3	Whereby a lack of knowledge is identified as preventing the improvement of horse welfare.
Understanding	3	Whereby a lack of understanding is identified as preventing the improvement of horse welfare.
Personal beliefs/cultural norms	3	References to people’s individual or group beliefs are a barrier to improving horse welfare.
Resistance to change/lack of trust	3	The concept of change or the communication of change communicated from/through a compromised source can act as a barrier to improving horse welfare through changing existing practices.
Financial constraints	3	Cited incidences where people’s financial situation prevents them from being able to operate differently to improve horse welfare.
Circumstances	3	Other personal circumstances (than financial) that impede delivering better horse welfare, often due to people being time poor.
Awareness	3	References to a lack of awareness preventing the improvement of horse welfare.
Lack of monitoring/reporting	3	References to how insufficient oversight of the horse population can prevent improvements to horse welfare.
Terminology/communications	3	This refers to how inappropriate language or messages can act as a barrier to improving horse welfare by making advice harder for all stakeholders to understand or adopt.
Indolence	3	References to caregiver laziness being a barrier to better horse welfare.
Personal bias	3	Distinct from personal beliefs, this refers to how people’s bias prevents them from accepting or being willing to accept the concept of making changes to benefit horse welfare, rather than their beliefs simply being at odds with recommended practices to better welfare standards.
Anthropomorphism	3	The issue of attempting to humanise horse behaviour or needs, resulting in a barrier to improving welfare; for example, resisting dieting advice because it makes an obese horse seem sad or not recognising dangerous behaviour because it is labelled as playing.
Lack of Clarity (comms and legislation)	3	This refers to how conflicting or variable language and guidance can impede horse welfare by confusing stakeholders.
Availability/consistency of training	3	As opposed to a lack of training, this cites how variable or inaccessible training for caregivers creates a barrier to better horse welfare.
Reactive strategies	3	Criticisms of how reliance on reactive strategies—e.g., punitive measures for lawbreakers or issuing advice after an incident—are a barrier to improving horse welfare.
Consumer demand	3	The pressure of the commercial horse market has been identified as a barrier to better horse welfare.
Lack of research	3	Some welfare concerns are poorly understood and as a result have been identified as hard to improve upon; thus, the lack of research in those areas is cited as a barrier to improving horse welfare.
Lack of tools	3	This refers to the limited available supportive resources for caregivers, for example accessible welfare assessment protocols to enable horse owners to identify areas for improvement.
Further research	4	Although not a solution in itself, more investigation and data into various welfare concerns, their causes or other contributing factors is suggested as necessary to improve horse welfare.
Increased communications	4	This includes increased awareness, meaning more widespread information about horse welfare issues and how to prevent them.
Culture/behaviour change	4	Referring to practical, tangible changes in the approaches of horse caregivers and stakeholders in the sector.
Education	4	Specifically structured learning and training, as opposed to passive information about sharing approaches.
Industry led initiatives/commitment	4	Refers to recommendations for improvements to horse welfare to stem from examples set at high levels of industry, essentially setting new norms from the top down.
Legislation	4	References to improved laws and legal guidelines as a solution to poor horse welfare.
Monitoring/reporting	4	Increased oversight of the horse population, improved registration and potential inspection of horses to reduce welfare concerns.
Updated training	4	New and improved training for horse owners and caregivers, in line with modern welfare standards and scientific findings.
Objective/holistic response	4	Refers to suggestions for whole system, widespread changes, i.e., a multilevel approach to reduce all kinds of welfare concerns, with both preventative and reactive measures.
Regulation/licensing	4	Distinct from legislation and monitoring/reporting, this refers to introducing/increasing requirements for ownership, facility provision, etc.
Financial support	4	References to creating sources of monetary support for organisations and for owners to ease pressures that compound welfare issues.
Reliable/robust assessment/tools	4	This category includes suggestions for new and improved welfare assessment methods, for the industry and for stakeholders at all levels to access and be able to use to better understand and, thus, improve horse welfare.
Prioritised resource	4	In opposition to a holistic response, this refers to suggestions to prioritise currently limited resources on tackling specific welfare issues and/or their causes.

**Table A4:** Definitions of Inputs from Search 2.

Input	Theme	Contributory vs. Outcome Definition
Increased understanding	5	Outcome Includes increased awareness and knowledge. Greater comprehension of animal welfare and related factors as a success of education,
Cultural change	5	Outcome A positive shift in general attitudes and beliefs as a result of education, cited as a success of education,
Institutional/national support	5	Contributor This refers to the need for high-level support (publicly and in terms of resource) for education to be successful,
WTP	5	Outcome Willingness (or ability) To Pay. This refers to how education can make people more likely or better able to pay more for better animal welfare outcomes.
Commitment to welfare	5	Outcome Increased dedication to improving animal welfare is cited as a success of education.
Interactive/demonstrative	5	Contributor Format of education favoured to increase the likelihood of a successful outcome.
Open to new ideas	5	Outcome Greater openness to concepts and beliefs other than those previously held cited as a success of education.
Interdisciplinary	5	Contributor Style of education favoured to increase the likelihood of a success outcome.
Attitude change	5	Outcome A shift in an individual’s or group’s approach to a set of ideas or practices, in this case towards more welfare friendly concepts, cited as a success of education.
Critical thinking	5	Outcome Increased ability to think analytically and objectively about information cited as a success of education.
Communication based/cohesive	5	Contributor Format of education favoured to increase the likelihood of a successful outcome.
Reduced animal welfare issues	5	Outcome A measurable decrease in animal welfare concerns or an increased level of good animal welfare as a result of education.
Increased skill	5	Outcome Heightened ability of participants when practicing taught processes as a result of education.
Appealing to stakeholders	5	Outcome A cited success of education is that it is positively received by stakeholders in a given industry, and potentially makes them view participants more favourably.
Targeted	5	Contributor Style of education favoured to increase the likelihood of a successful outcome.
Multilevel	5	Contributor Format of education favoured to increase the likelihood of a successful outcome.
Increased production/efficiency/safety	5	Outcome Improved performance along a variety of measures as a direct result of education.
Online	5	Contributor Style of education favoured to increase the likelihood of a successful outcome.
Voluntary	5	Contributor As opposed to mandatory, education being chosen by the participant is cited as more successful/impactful.
Consequence focussed/animal centred	5	Contributor Design of education favoured to increase the likelihood of a successful outcome.
Assessed	5	Contributor Format of education favoured to increase the likelihood of a successful outcome.
Unclear effect on behaviour	6	Outcome References to a lack of or inconsistent measure of behaviour change as a result of education, cited as a limitation of educational interventions.
Availability	6	Contributor Limited provision or accessibility of education is cited as a limitation of possible successful outcomes.
Other circumstances	6	Outcome Includes socio-economic factors of age, gender, home environment, etc. Cited as a potential limitation for education to produce successful outcomes, in spite of good intentions or improved knowledge.
Syllabus content	6	Contributor References to education design and materials being a limiting factor in producing successful outcomes.
Resistance	6	Outcome Particularly noted for mandatory education, this refers to education having a limited influence over beliefs if people are set against the concepts taught from the outset or discover that new ideas are too opposed to their existing ideals.
Perception/prioritisation	6	Outcome References to education’s limited influence due to people’s attitudes towards education in principle.
Resource	6	Contributor Referring to the cost of implementation as well as the time and availability of trained persons to deliver education, a lack of resource is cited as a limiting factor for education.
Lack of consensus on delivery	6	Contributor Disagreement over how to deliver education—note the wide range of suggestions under Theme 5—is cited as leading to limited success of developing and implementing programmes.
Tailoring/discipline specific	6	Contributor Refers to the limitations of delivering education specifically for small target audiences, whereby there are too many education programmes as they are all built for specific purposes (and may differ on key points), or education becomes too granular to be widely applicable.
Communication	6	Contributor Insufficient or poorly designed messages around education limit its effectiveness.
Reduced perspective taking	6	Outcome Refers to instances where participants appear less likely or able to see other perspectives as a result of following educational recommendations.
Lack of uptake	6	Outcome Low levels of participation within a population are cited to limit the overall effectiveness of the programme.
Needs further research	6	Contributor References to more investigation being required in order to develop a successful educational programme.
Language	6	Contributor Refers to the negative impact of using the wrong type of language for the target audience.
Theoretical	6	Contributor Format of education suggested as reducing the likelihood of successful outcomes.
Generalisation	6	Contributor As opposed to tailored/discipline specific, this refers to the limitations of too widely focussed or overly generic education.
Lack of improved skill	6	Outcome Refers to instances where education was not deemed to have produced an increase in ability, despite potentially increasing knowledge.
Farm-based	6	Contributor Format of education suggested as less likely to lead to a successful outcome.
Consumer tool	7	Outcome Refers to licensing being positively used to inform consumer decisions in favour of more animal welfare friendly choices.
Monitoring/regulation	7	Outcome Increased oversight of a population cited as a success pf licensing.
Minimum standards	7	Outcome Maintaining and upholding basic guideline standards for compliance cited as a success of licensing.
Education (support)	7	Contributor Being supported by education is cited as making licensing more likely to be effective for improving animal welfare.
National support	7	Contributor National level endorsement by a recognised body, or example led participation by key high-level groups, cited as making licensing more likely to be successful.
Financial support	7	Contributor Cited as increasing the successfulness of licensing.
Culture/behaviour change	7	Outcome Refers to a measurable positive shift in attitudes or practices as a direct result of licensing.
Inclusive	7	Contributor Refers to the need for licensing programmes not to disadvantage any particular groups in order to be successful and/or positively received.
Public support/trust	7	Contributor Trust refers to the need for a receptive public attitude towards the body implementing licensing, their communications and the rationale for licensing itself. Positive attitudes towards a licensing scheme are cited as a requirement for success.
Generates income	7	Outcome Increased profit (or decreased losses) cited as a success of licensing.
Improved welfare	7	Outcome More positive animal welfare (or less welfare concerns) measured as a direct result of licensing.
Self-motivated	7	Contributor Refers to the increased likelihood of licensing to produce a positive outcome if the licensee has an individual, voluntary drive to obtain the license.
Active participation	7	Contributor Active engagement with a licensing scheme and its goals is cited as increasing the likelihood of success.
Enforcement	7	Contributor It is cited that for licensing to be successful, especially when mandatory, then participation and maintenance need to be monitored and action taken if licensing lapses or is neglected.
Collaborative	7	Contributor Design of licensing scheme, which is suggested as increasing the likelihood of successful outcomes.
Enforcement	8	Contributor Refers to the necessity for licensing to be strictly monitored and upheld in order to be effective, this is cited as a limitation of licensing as the license itself is not necessarily producing positive outcomes, and enforcement is resource heavy.
Resource	8	Contributor The high cost and administration required to implement a licensing programme is cited as a limitation.
Assumptions/clarity	8	Contributor Relating to animal welfare. This refers to the limited effect of licensing due to varied or muddled understandings of welfare and the potential for licensing schemes to assume a shared understanding.
Uptake	8	Outcome Lack of participation is cited as a limitation for voluntary licensing schemes.
Competition	8	Outcome Industrial and personal level, the need to perform highly can outweigh the aims of a licensing scheme, reducing the effectiveness.
Lack of trust	8	Outcome As well as trust being required for success, a lack of it from the target group can inevitably limit the effectiveness of licensing. Equally, licensing itself may reduce the trust of the public in the licenser if the implementation is seen as curtailing or checking day to day activities.
Professional/industrial focus	8	Contributor An aim or rationale for licensing cited as limiting the successful outcomes.
Restrictive	8	Outcome Refers to instances where licensing limits practices or generates exclusivity of a group, cited as limiting the effectiveness of licensing.
Administration	8	Contributor The need for either local or centralised administration is cited as a limiting factor for effective licensing, related to resources but specifically highlighting the additional challenges of such organisation.
Limited influence on behaviour	8	Outcome Lack of a measurable shift in practices as a result of licensing cited as a limitation of its effectiveness.
Reduced innovation	8	Outcome Related to “restrictive”, this specifically refers to instances where licensing has hindered the generation/implementation/sharing of new ideas or practices.
Lack of welfare focus	8	Contributor Insufficient prioritisation of animal welfare needs within the licensing requirements limits the efficacy of licensing to tackle welfare concerns.
Minimum standards only	8	Contributor This refers to the limitation of licensing, which is designed to only maintain minimum standards, as opposed to holding licensees to higher standards of animal welfare. The minimum standards approach is critiqued as sending the wrong message.
Communication	8	Contributor Advertising and explanation of licensing programmes, either lacking or not appropriately targeted, is cited as reducing the likelihood of uptake or success.
Transparency	8	Contributor A lack of clear standards or lack of openness to the public are cited as limiting factors for licensing to be likely to succeed.
Stress	8	Outcome A negative potential outcome of licensing.
Political support	8	Contributor This has been identified by some as a requirement for licensing to be implemented.
Gaps in industry	8	Contributor Licensing for specific groups may expose, overlook or exploit pockets within a given sector, causing new issues rather than providing overall positive outcomes.
Variability	8	Contributor Different schemes offer different guidelines and potential incentives, this may limit the efficacy of licensing especially when voluntary or when licensees compare voluntary against mandatory standards.

## Appendix C

This appendix contains a complete list of all included papers from both Searches. * Denotes duplicates in Search 2 from Search 1. ** Signifies papers that, upon review, may have been included in error due to revised criteria or additional time to cross check the consistency of the data collection across both searches.

**Table A5:** Papers included in the data set from Search 1 and Search 2. * Denotes duplicates in Search 2 from Search 1. ** Signifies papers which, upon review, may have been excluded or included with revised criteria or additional time to cross check the consistency of the data collection across both searches.

Included in Search 1	Included in Search 2
** Adams, C., Arratoon, C., Boucher, J., Cartier, G., Chalmers, D., Anne Dell, C., Dell, D., Dryka, D., Duncan, R., Dunn, K. and Hopkins, C., 2015. The helping horse: How equine assisted learning contributes to the wellbeing of First Nations youth in treatment for volatile substance misuse. Human-animal interaction bulletin, (2015).	Almeida, A. et al. (2017) Children’s Opinions about Zoos: A Study of Portuguese and Spanish Pupils. Anthrozoös. [Online] 30 (3), 457–472.
Anttila, M. et al. (2022) Oral Dimensions Related to Bit Size in Adult Horses and Ponies. Frontiers in veterinary science. [Online] 9.	Almeida, A. and Garcia Fernandez, B., 2021. Attitudes towards animal welfare in Portuguese students from the 6th and the 9th year of schooling: Implications for environmental education. Environmental Education Research, 27(6), pp. 911–935.
Baumgartner, M. et al. (2015) Lying behaviour of group-housed horses in different designed areas with rubber mats, shavings and sand bedding. Pferdeheilkunde Equine Medicine. [Online] 31 (3), 211–220.	Armitt, K.-A. et al. (2023) A Qualitative Analysis of Management Perspectives on Seeking to Implement the Foster Cat Project in Residential Aged Care in the Context of COVID-19. International journal of environmental research and public health. [Online] 20 (1).
Björnsdóttir, S. et al. (2014) Bit-related lesions in Icelandic competition horses. Acta veterinaria Scandinavica: consilio Societatum Veterinarium Scandinavicarum edita. [Online] 56 (1).	Baird, B. A. et al. (2016) Program animal welfare: Using behavioral and physiological measures to assess the well-being of animals used for education programs in zoos. Applied animal behaviour science. [Online] 176150–162.
Bornmann, T. et al. (2021) Investigating Equestrians’ Perceptions of Horse Happiness: An Exploratory Study. Journal of equine veterinary science. [Online] 104.	Bejaei, M. et al. (2011) Influences of demographic characteristics, attitudes, and preferences of consumers on table egg consumption in British Columbia, Canada. Poultry science. [Online] 90 (5), 1088–1095.
Box, J. R. et al. (2020) Insulin dysregulation in a population of Finnhorses and associated phenotypic markers of obesity. Journal of veterinary internal medicine. [Online] 34 (4), 1599–1605.	Bianchi, F. et al. (2018) Interventions targeting conscious determinants of human behaviour to reduce the demand for meat: a systematic review with qualitative comparative analysis. The international journal of behavioral nutrition and physical activity. [Online] 15 (1),
Bradshaw, J.W.S. and Casey, R.A., 2007. Anthropomorphism and anthropocentrism as influences in the quality of life of companion animals. Animal welfare, 16(S1), pp. 149–154.	Bir, C. et al. (2019) US Residents’ Perceptions of Dog Welfare Needs and Canine Welfare Information Sources. Journal of applied animal welfare science. [Online] 22 (1), 42–68.
Brinkmann, L. et al. (2013) Effect of long-term feed restriction on the health status and welfare of a robust horse breed, the Shetland pony (*Equus ferus caballus*). Research in veterinary science. [Online] 94 (3), 826–831.	Boaitey, A. & Minegishi, K. (2020) Who are farm animal welfare conscious consumers? British Food Journal. [Online] 122 (12), 3779–3796.
Burla, J.-B. et al. (2017) Space Allowance of the Littered Area Affects Lying Behavior in Group-Housed Horses. Frontiers in veterinary science. [Online] 4.	Bozzo, G. (2019) Consumer attitudes towards animal welfare and their willingness to pay. Veterinaria italiana. [Online] 55 (4), 289–297.
Campbell, M. L. H. (2021) An Ethical Framework for the Use of Horses in Competitive Sport: Theory and Function. Animals: an open access journal from MDPI. [Online] 11 (6).	Bruckner, H. K. & Kowasch, M. (2019) Moralizing meat consumption: Bringing food and feeling into education for sustainable development. Policy futures in education. [Online] 17 (7), 785–804.
Carroll, S. L. et al. (2020) An online survey investigating perceived prevalence and treatment options for stereotypic behaviours in horses and undesirable behaviours associated with handling and riding. Equine veterinary education. [Online] 32 (S11), 71–81.	Buddle, E.A., Bray, H.J. and Ankeny, R.A., 2021. “Of course we care!“: A qualitative exploration of Australian livestock producers’ understandings of farm animal welfare issues. Journal of Rural Studies, 83, pp. 50–59.
Carroll, S. L. et al. (2022) Moving toward Fear-Free Husbandry and Veterinary Care for Horses. Animals: an open access journal from MDPI. [Online] 12 (21).	Carpenter, A. F. & Song, W. (2016) Changing Attitudes about the Weak: Social and Legal Conditions for Animal Protection in China. Critical Asian studies. [Online] 48 (3), 380–399.
Čebulj-Kadunc, N. et al. (2022) Fluctuations of Physiological Variables during Conditioning of Lipizzan Fillies before Starting under Saddle. Animals: an open access journal from MDPI. [Online] 12 (7).	Celozzi, S., Battini, M., Prato-Previde, E. and Mattiello, S., 2022. Humans and goats: Improving knowledge for a Better Relationship. Animals, 12(6), p. 774.
Cerasoli, F. et al. (2022) Assessment of Welfare in Groups of Horses with Different Management, Environments and Activities by Measuring Cortisol in Horsehair, Using Liquid Chromatography Coupled to Hybrid Orbitrap High-Resolution Mass Spectrometry. Animals: an open access journal from MDPI. [Online] 12 (14).	Charlebois, S. et al. (2016) Meat consumption and higher prices. British Food Journal. [Online] 118 (9), 2251–2270.
Chan, M. C.-H. et al. (2022) Human–Animal Interactions and the Promotion of Social and Emotional Competencies: A Scoping Review. Anthrozoös. [Online] 35 (5), 647–692.	Clark, B. et al. (2016) A Systematic Review of Public Attitudes, Perceptions and Behaviours Towards Production Diseases Associated with Farm Animal Welfare. Journal of agricultural and environmental ethics. [Online] 29 (3), 455–478.
Chapouthier, G. (2010) Human Direct Actions May Alter Animal Welfare, a Study on Horses (*Equus caballus*). PloS one. [Online] 5 (4).	Collins, C. et al. (2019) Zoological education: Can it change behaviour? Applied animal behaviour science. [Online] 220.
** Coffin, J., 2019. The Nguudu Barndimanmanha Project-improving social and emotional wellbeing in aboriginal youth through equine assisted learning. Frontiers in public health, 7, p. 278.	* COLLINS, J. A. et al. (2010) Evaluation of current equine welfare issues in Ireland: Causes, desirability, feasibility and means of raising standards. Equine veterinary journal. [Online] 42 (2), 105–113.
COLLINS, J. A. et al. (2010) Evaluation of current equine welfare issues in Ireland: Causes, desirability, feasibility and means of raising standards. Equine veterinary journal. [Online] 42 (2), 105–113.	* Collins, J. et al. (2008) The structure and regulation of the Irish equine industries: Links to considerations of equine welfare. Irish veterinary journal: journal of the Irish Veterinary Association = Iris Treidliachta Eireann. [Online] 61 (11), 746–756.
Collins, J. et al. (2008) The structure and regulation of the Irish equine industries: Links to considerations of equine welfare. Irish veterinary journal: journal of the Irish Veterinary Association = Iris Treidliachta Eireann. [Online] 61 (11), 746–756.	de Boer, J. & Aiking, H. (2022) EU Citizen support for climate-friendly agriculture (Farm) and dietary options (Fork) across the left-right political spectrum. Climate policy. [Online] 1–13.
Collins, J., Hanlon, A., More, S.J., Wall, P.G. and Duggan, V., 2009. Policy Delphi with vignette methodology as a tool to evaluate the perception of equine welfare. The Veterinary Journal, 181(1), pp. 63–69.	De Briyne, N. et al. (2018) ‘Phasing out pig tail docking in the EU—present state, challenges and possibilities’. Porcine health management. [Online] 4 (1).
Collins, J.A., More, S.J., Hanlon, A., Wall, P.G., McKenzie, K. and Duggan, V., 2012. Use of qualitative methods to identify solutions to selected equine welfare problems in Ireland. Veterinary Record, 170(17), pp. 442–442.	de Jonge, J. et al. (2015) Different shades of grey: Compromise products to encourage animal friendly consumption. Food quality and preference. [Online] 4587–99.
Contalbrigo, L. et al. (2021) Equine-Assisted Interventions (EAIs) for Children with Autism Spectrum Disorders (ASD): Behavioural and Physiological Indices of Stress in Domestic Horses (*Equus caballus*) during Riding Sessions. Animals: an open access journal from MDPI. [Online] 11 (6).	de Mori, B. et al. (2019) A Protocol for the Ethical Assessment of Wild Animal–Visitor Interactions (AVIP) Evaluating Animal Welfare, Education, and Conservation Outcomes. Animals: an open access journal from MDPI. [Online] 9 (8).
Cook, W. R. & Kibler, M. (2019) Behavioural assessment of pain in 66 horses, with and without a bit. Equine veterinary education. [Online] 31 (10), 551–560.	de Oliveira Padilha, L. G. et al. (2022) Consumers’ attitudes towards lab-grown meat, conventionally raised meat and plant-based protein alternatives. Food quality and preference. [Online] 99.
Dalla Costa, E. et al. (2017) Initial outcomes of a harmonized approach to collect welfare data in sport and leisure horses. Animal: an international journal of animal bioscience. [Online] 11 (2), 254–260.	Dentoni, D. et al. (2014) Disentangling direct and indirect effects of credence labels. British Food Journal. [Online] 116 (6), 931–951.
Dalla Costa, E. et al. (2019) Text Mining Analysis to Evaluate Stakeholders’ Perception Regarding Welfare of Equines, Small Ruminants, and Turkeys. Animals: an open access journal from MDPI. [Online] 9 (5).	Denver, S. et al. (2021) Consumer preferences for reduced antibiotic use in Danish pig production. Preventive veterinary medicine. [Online] 189.
Douglas, J. et al. (2022) Social Licence to Operate: What Can Equestrian Sports Learn from Other Industries? Animals: an open access journal from MDPI. [Online] 12 (15).	Diana, A. et al. (2021) Why do Irish pig farmers use medications? Barriers for effective reduction of antimicrobials in Irish pig production. Irish veterinary journal: journal of the Irish Veterinary Association = Iris Treidliachta Eireann. [Online] 74 (1).
DuBois, C. et al. (2018) Examining Canadian Equine Industry Participants’ Perceptions of Horses and Their Welfare. Animals: an open access journal from MDPI. [Online] 8 (11).	* DuBois, C. et al. (2018) Examining Canadian Equine Industry Participants’ Perceptions of Horses and Their Welfare. Animals: an open access journal from MDPI. [Online] 8 (11).
DuBois, C., Hambly Odame, H., Haley, D.B. and Merkies, K., 2018. An exploration of industry expert perception of Canadian equine welfare using a modified Delphi technique. PloS One, 13(7), p.e0201363.	Dunne, C. & Siettou, C. (2020) UK consumers’ willingness to pay for laying hen welfare. British Food Journal. [Online] 122 (9), 2867–2880.
DuBois, C., Hambly-Odame, H., Haley, D.B. and Merkies, K., 2017. An exploration of industry expert perception of equine welfare using vignettes. Animals, 7(12), p. 102.	Ekakoro, J. E. et al. (2019) Drivers, alternatives, knowledge, and perceptions towards antimicrobial use among Tennessee beef cattle producers: a qualitative study. BMC veterinary research. [Online] 15 (1).
Erasmus, M. & Rollins, J. (2022) Visitors’ Self-Reported Knowledge and Attitudes about an Animal-Free Exhibit on Animal Welfare. Journal of applied animal welfare science. [Online] 25 (4), 382–395.	Ellingsen, K. et al. (2015) Who cares about fish welfare? British Food Journal. [Online] 117 (1), 257–273.
Everett, J. B. (2018) Effects of stacked wedge pads and chains applied to the forefeet of Tennessee Walking Horses for a five-day period on behavioral and biochemical indicators of pain, stress, and inflammation. American journal of veterinary research. 79 (1), 21–32.	Elwin, A. et al. (2020) On the Record: An Analysis of Exotic Pet Licences in the UK. Animals: an open access journal from MDPI. [Online] 10 (12).
Fenner, K., Mclean, A.N. and McGreevy, P.D., 2019. Cutting to the chase: How round-pen, lunging, and high-speed liberty work may compromise horse welfare. Journal of Veterinary Behavior, 29, pp.88–94.	Enlund, K. B. et al. (2020) Dental home care in dogs—a questionnaire study among Swedish dog owners, veterinarians and veterinary nurses. BMC veterinary research. [Online] 16 (1).
Fiedler, J. & McGreevy, P. (2016) Reconciling Horse Welfare, Worker Safety, and Public Expectations: Horse Event Incident Management Systems in Australia. Animals: an open access journal from MDPI. [Online] 6 (3).	Erasmus, M. & Rollins, J. (2022) Visitors’ Self-Reported Knowledge and Attitudes about an Animal-Free Exhibit on Animal Welfare. Journal of applied animal welfare science. [Online] 25 (4), 382–395.
Fiedler, J., Thomas, M. and Ames, K., 2019, August. Informing a social license to operate communication framework: Attitudes to sport horse welfare. In Proceedings of the 15th International Society of Equitation Science Conference, Guelph, ON, Canada (pp. 19–21).	Estévez-Moreno, L. X. et al. (2021) Attitudes of meat consumers in Mexico and Spain about farm animal welfare: A cross-cultural study. Meat Science. [Online] 173.
Fine, A. H. & Andersen, S. J. (2021) A Commentary on the Contemporary Issues Confronting Animal Assisted and Equine Assisted Interactions. Journal of equine veterinary science. [Online] 100.	Fennell, D. A. et al. (2022) An animal welfare syllabus for wildlife tourism. Journal of sustainable tourism. [Online] 1–19.
Flauger, B. (2013) Aggression level and enclosure size in horses (*Equus caballus*). PFERDEHEILKUNDE. 29 (4), 495–+.	Fernandez, E. J. et al. (2009) Animal–visitor interactions in the modern zoo: Conflicts and interventions. Applied animal behaviour science. [Online] 120 (1–2), 1–8.
Furtado, T. et al. (2021) How Happy Are Equine Athletes? Stakeholder Perceptions of Equine Welfare Issues Associated with Equestrian Sport. Animals: an open access journal from MDPI. [Online] 11 (11),	Fletcher, K. et al. (2021) Contemplating the Five Domains model of animal welfare assessment: UK horse-owner perceptions of equine well-being. Animal welfare. [Online] 30 (3), 259–268.
Furtado, T., Perkins, E., Pinchbeck, G., McGowan, C., Watkins, F. and Christley, R., 2022. Exploring human behavior change in equine welfare: Insights from a COM-B analysis of the UK’s equine obesity epidemic. Frontiers in Veterinary Science, 9, p. 961537.	Fourage, A. et al. (2022) It’s a sign: Animal welfare and zoo type are predictors of animal identification signage usage and quality at zoo exhibits. Zoo biology. [Online]
Gehlen, H. et al. (2021) Keeping Stallions in Groups—Species-Appropriate or Relevant to Animal Welfare? Animals: an open access journal from MDPI. [Online] 11 (5).	Hagevoort, G. R. et al. (2013) A Review of Health and Safety Leadership and Managerial Practices on Modern Dairy Farms. Journal of agromedicine. [Online] 18 (3), 265–273.
Gorgasseri, I. (2007) Faecal cortisol metabolites in Quarter Horses during initial training under field conditions. Wiener Tierärztliche Monatsschrift. 94 (9–10), 226–230.	Harwood, W. & Drake, M. (2020) The influence of automatic associations on preference for milk type. Journal of dairy science. [Online] 103 (12), 11218–11227.
Greening, L. and McBride, S., 2022. A Review of Equine Sleep: Implications for Equine Welfare. Frontiers in Veterinary Science, 9, p. 916737.	Hawkins, R.D., Williams, J.M. and Scottish Society for the Prevention of Cruelty to Animals, 2017. Assessing effectiveness of a nonhuman animal welfare education program for primary school children. Journal of Applied Animal Welfare Science, 20(3), pp. 240–256.
Griffin, C. J. (2012) Animal maiming, intimacy and the politics of shared life: the bestial and the beastly in eighteenth- and early nineteenth-century England. Transactions/. [Online] 37 (2), 301–316.	He, J. et al. (2020) A review of research on plant-based meat alternatives: Driving forces, history, manufacturing, and consumer attitudes. Comprehensive reviews in food science and food safety. [Online] 19 (5), 2639–2656.
Guarneros, M. et al. (2020) The underexplored role of chemical communication in the domestic horse, *Equus caballus*. Journal of veterinary behavior: clinical applications and research: official journal of: Australian Veterinary Behaviour Interest Group, International Working Dog Breeding Association. [Online] 3889–95.	Hempstead, M. N. et al. (2021) Health and Welfare Survey of 30 Dairy Goat Farms in the Midwestern United States. Animals: an open access journal from MDPI. [Online] 11 (7).
Hartmann, E. et al. (2012) Keeping horses in groups: A review. Applied animal behaviour science. [Online] 136 (2–4), 77–87.	Hillmann, E. (2016) What Difference Does a Visit Make? Changes in Animal Welfare Perceptions after Interested Citizens Tour a Dairy Farm. PloS one. [Online] 11 (5).
Hausberger, M. et al. (2012) On the significance of adult play: what does social play tell us about adult horse welfare? The Science of nature = Naturwissenschaften. [Online] 99 (4), 291–302.	Hillmann, E. (2019) Positive attitudes, positive outcomes: The relationship between farmer attitudes, management behaviour and sheep welfare. PloS one. [Online] 14 (7).
Hausberger, M., Stomp, M., Sankey, C., Brajon, S., Lunel, C. and Henry, S., 2019. Mutual interactions between cognition and welfare: The horse as an animal model. Neuroscience & Biobehavioral Reviews, 107, pp. 540–559.	Hoerterer, C. et al. (2022) Informed choice: The role of knowledge in the willingness to consume aquaculture products of different groups in Germany. Aquaculture. [Online] 556.
Hawson, L.A., McLean, A.N. and McGreevy, P.D., 2010. Variability of scores in the 2008 Olympic dressage competition and implications for horse training and welfare. Journal of Veterinary Behavior, 5(4), pp.170–176.	Illmann, G. et al. (2014) Mapping farm animal welfare education at university level in Europe. Animal welfare. [Online] 23 (4), 401–410.
** Heleski, C. R. & Anthony, R. (2012) Science alone is not always enough: The importance of ethical assessment for a more comprehensive view of equine welfare. Journal of veterinary behavior: clinical applications and research: official journal of: Australian Veterinary Behaviour Interest Group, International Working Dog Breeding Association. [Online] 7 (3), 169–178.	Jamieson, J., Reiss, M.J., Allen, D., Asher, L., Wathes, C.M. and Abeyesinghe, S.M., 2012. Measuring the success of a farm animal welfare education event. Animal Welfare, 21(1), pp. 65–75.
** Heleski, C. R. & Zanella, A. J. (2006) Animal science student attitudes to farm animal welfare. Anthrozoös. [Online] 19 (1), 3–16.	Jo, H. et al. (2022) A Survey of Broiler Farmers’ Perceptions of Animal Welfare and their Technical Efficiency: A Case Study in Northeast China. Journal of applied animal welfare science. [Online] 25 (3), 275–286.
Hemsworth, L. M. et al. (2015) Recreational horse welfare: The relationships between recreational horse-owner attributes and recreational horse welfare. Applied animal behaviour science. [Online] 1651–16.	Jones, M. et al. (2012) Food sustainability education as a route to healthier eating: evaluation of a multi-component school programme in English primary schools. Health education research. [Online] 27 (3), 448–458.
Hill, E. et al. (2015) Apparatus use in popular equestrian disciplines in Australia. Journal of veterinary behavior: clinical applications and research: official journal of: Australian Veterinary Behaviour Interest Group, International Working Dog Breeding Association. [Online] 10 (2), 147–152.	Kang, O.-D. & Lee, W.-S. (2016) Changes in Salivary Cortisol Concentration in Horses during Different Types of Exercise. Asian-Australasian journal of animal sciences. [Online] 29 (5), 747–752.
Hitchens, P. et al. (2017) An epidemiological analysis of equine welfare data from regulatory inspections by the official competent authorities. Animal: an international journal of animal bioscience. [Online] 11 (7), 1237–1248.	Klöckner, C. A. et al. (2022) Milk, Meat, and Fish From the Petri Dish—Which Attributes Would Make Cultured Proteins (Un)attractive and for Whom? Results From a Nordic Survey. Frontiers in sustainable food systems. [Online] 6.
Hitchens, P. L. et al. (2016) Prevalence and risk factors for overweight horses at premises in Sweden assessed using official animal welfare control data. Acta veterinaria Scandinavica: consilio Societatum Veterinarium Scandinavicarum edita. [Online] 58 (S1),	Kopnina, H. & Cherniak, B. (2015) Cultivating a Value for Non-Human Interests through the Convergence of Animal Welfare, Animal Rights, and Deep Ecology in Environmental Education. Education sciences. [Online] 5 (4), 363–379.
Hockenhull, J. & Creighton, E. (2015) The day-to-day management of UK leisure horses and the prevalence of owner-reported stable-related and handling behaviour problems. Animal welfare. [Online] 24 (1), 29–36.	Kopnina, H. (2018) Attitudes of the public towards halal food and associated animal welfare issues in two countries with predominantly Muslim and non-Muslim populations. PloS one. [Online] 13 (10).
Hockenhull, J. and Furtado, T., 2021. Escaping the gilded cage: Could COVID-19 lead to improved equine welfare? A review of the literature. Applied Animal Behaviour Science, 237, p. 105303.	Kraak, V. I. (2022) Perspective: Unpacking the Wicked Challenges for Alternative Proteins in the United States: Can Highly Processed Plant-Based and Cell-Cultured Food and Beverage Products Support Healthy and Sustainable Diets and Food Systems? Advances in nutrition. [Online] 13 (1), 38–47.
Hogg, R. C. & Hodgins, G. A. (2021) Symbiosis or Sporting Tool? Competition and the Horse-Rider Relationship in Elite Equestrian Sports. Animals: an open access journal from MDPI. [Online] 11 (5),	Kupsala, S. et al. (2015) Citizen Attitudes to Farm Animals in Finland: A Population-Based Study. Journal of agricultural and environmental ethics. [Online] 28 (4), 601–620.
Holcomb, K. E. et al. (2010) Unwanted horses: The role of nonprofit equine rescue and sanctuary organizations1. Journal of animal science. [Online] 88 (12), 4142–4150.	Kwasny, T. et al. (2022) Towards reduced meat consumption: A systematic literature review of intervention effectiveness, 2001–2019. Appetite. [Online] 168.
Holmes, T. Q. & Brown, A. F. (2022) Champing at the Bit for Improvements: A Review of Equine Welfare in Equestrian Sports in the United Kingdom. Animals: an open access journal from MDPI. [Online] 12 (9).	Lazíková, J., Bandlerová, A., Rumanovská, Ľ., Takáč, I. and Lazíková, Z., 2019. Crop diversity and common agricultural policy—the case of Slovakia. Sustainability, 11(5), p.1416.
Horseman, S. V. et al. (2017) Equine Welfare in England and Wales: Exploration of Stakeholders’ Understanding. Journal of applied animal welfare science. [Online] 20 (1), 9–23.	Liang, Y. et al. (2022) Consumer Preferences for Animal Welfare in China: Optimization of Pork Production-Marketing Chains. Animals: an open access journal from MDPI. [Online] 12 (21).
Horseman, S.V., Buller, H., Mullan, S. and Whay, H.R., 2016. Current welfare problems facing horses in Great Britain as identified by equine stakeholders. PLoS One, 11(8), p.e0160269.	Liang, Y. et al. (2023) Emerging market for pork with animal welfare attribute in China: An ethical perspective. Meat Science. [Online] 195.
Houpt, K. A. (2012) Motivation for cribbing by horses. Animal welfare. 21 (1), 1–7.	Lorenz, I. (2021) Calf health from birth to weaning—an update. Irish veterinary journal: journal of the Irish Veterinary Association = Iris Treidliachta Eireann. [Online] 74 (1).
Jones McVey, R., 2021. An Ethnographic Account of the British Equestrian Virtue of Bravery, and Its Implications for Equine Welfare. Animals, 11(1), p. 188.	Losada-Espinosa, N. et al. (2020) Stockpeople and Animal Welfare: Compatibilities, Contradictions, and Unresolved Ethical Dilemmas. Journal of agricultural and environmental ethics. [Online] 33 (1), 71–92.
Kau, S. et al. (2020) Bit type exerts an influence on self-controlled rein tension in unridden horses. Scientific reports. [Online] 10 (1).	Luebke, J. F. et al. (2016) Zoo Visitors’ Affective Responses to Observing Animal Behaviors. Visitor studies. [Online] 19 (1), 60–76.
Kelly, K.J., McDuffee, L.A. and Mears, K., 2021. The effect of human–horse interactions on equine behaviour, physiology, and welfare: A scoping review. animals, 11(10), p. 2782.	Lyles, J. L. & Calvo-Lorenzo, M. S. (2014) BILL E. KUNKLE INTERDISCIPLINARY BEEF SYMPOSIUM: Practical developments in managing animal welfare in beef cattle: What does the future hold?1. Journal of animal science. [Online] 92 (12), 5334–5344.
König v. Borstel, U., 2013. Assessing and influencing personality for improvement of animal welfare: a review of equine studies. CABI Reviews, (2013), pp. 1–27.	MacKay, J. R. D. (2020) Discipline-Based Education Research for Animal Welfare Science. Frontiers in veterinary science. [Online] 7.
Lansade, L. et al. (2019) Horse’s emotional state and rider safety during grooming practices, a field study. Applied animal behaviour science. [Online] 21743–47.	MacKay, J. R. et al. (2016) Massive Open Online Courses as a Tool for Global Animal Welfare Education. Journal of veterinary medical education. [Online] 43 (3), 287–301.
Leliveld, L. M. et al. (2013) The emergence of emotional lateralization: Evidence in non-human vertebrates and implications for farm animals. Applied animal behaviour science. [Online] 145 (1–2), 1–14.	Main, D. & Mullan, S. (2012) Economic, education, encouragement and enforcement influences within farm assurance schemes. Animal welfare. [Online] 21 (S1), 107–111.
Lesimple, C. et al. (2016) How to keep your horse safe? An epidemiological study about management practices. Applied animal behaviour science. [Online] 181105–114.	Martelli, G. (2009) Consumers’ perception of farm animal welfare: an Italian and European perspective. Italian journal of animal science: journal and official organ of the Scientific Association of Animal Production (A.S.P.A.). [Online] 8 (sup1), 31–41.
Lofgren, E.A., Rice, B.M. and Brady, C.M., 2022. Exploring perceptions of equine welfare scenarios using a positive approach. Journal of Applied Animal Welfare Science, 25(1), pp. 54–61.	May, C. & Previte, J. (2016) Understanding the midstream environment within a social change systems continuum. Journal of social marketing. [Online] 6 (3), 258–276.
Luke, K. et al. (2022) A new approach to horse welfare based on systems thinking. Animal welfare. [Online] 31 (1), 37–49.	Maynard, L. (2018) Media Framing of Zoos and Aquaria: From Conservation to Animal Rights. Environmental communication. [Online] 12 (2), 177–190.
Luke, K. L. et al. (2022) New insights into ridden horse behaviour, horse welfare and horse-related safety. Applied animal behaviour science. [Online] 246.	McCarthy, B. L. (2015) Trends in Organic and Green Food Consumption in China: Opportunities and Challenges for Regional Australian Exporters. Journal of economic and social policy. 17 (1).
Luke, K.L., McAdie, T., Warren-Smith, A.K. and Smith, B.P., 2023. Bit use and its relevance for rider safety, rider satisfaction and horse welfare in equestrian sport. Applied Animal Behaviour Science, 259, p. 105855.	* McKendree, M. G. S. et al. (2014) BIOETHICS SYMPOSIUM II: Current factors influencing perceptions of animals and their welfare1. Journal of animal science. [Online] 92 (5), 1821–1831.
Luke, K.L., McAdie, T., Warren-Smith, A.K. and Smith, B.P., 2023. Untangling the Complex Relationships between Horse Welfare, Rider Safety, and Rider Satisfaction. Anthrozoös, pp. 1–16.	Mee, G. et al. (2022) Owner demographic factors are associated with suitable pet rabbit housing provision in the United Kingdom. The Veterinary record. [Online] 190 (12).
Luke, K.L., McAdie, T., Warren-Smith, A.K., Rawluk, A. and Smith, B.P., 2023. Does a Working Knowledge of Learning Theory Relate to Improved Horse Welfare and Rider Safety?. Anthrozoös, pp. 1–17.	Mellor, D. J. & Bayvel, A. C. D. (2008) New Zealand’s inclusive science-based system for setting animal welfare standards. Applied animal behaviour science. [Online] 113 (4), 313–329.
Luna, D. & Tadich, T. (2019) Why Should Human-Animal Interactions Be Included in Research of Working Equids’ Welfare? Animals: an open access journal from MDPI. [Online] 9 (2).	Mench, J. A. (2008) Farm animal welfare in the U.S.A.: Farming practices, research, education, regulation, and assurance programs. Applied animal behaviour science. [Online] 113 (4), 298–312.
Lundmark Hedman, F. et al. (2022) Swedish Trotting Horse Trainers’ Perceptions of Animal Welfare Inspections from Public and Private Actors. Animals: an open access journal from MDPI. [Online] 12 (11).	Menozzi, D. et al. (2021) Insects as Feed for Farmed Poultry: Are Italian Consumers Ready to Embrace This Innovation? Insects. [Online] 12 (5).
Lynch, M.J. and Genco, L.J., 2021. Cruelty against Animals’ Welfare (CAAW) Violations: a Study of Animal Welfare Act and Horse Welfare Act Enforcement Actions in the US, 2010–2014. Sociological Spectrum, 41(3), pp. 255–272.	Merlino, V. M. et al. (2022) Are Local Dairy Products Better? Using Principal Component Analysis to Investigate Consumers’ Perception towards Quality, Sustainability, and Market Availability. Animals: an open access journal from MDPI. [Online] 12 (11).
Mach, N. et al. (2021) Gut microbiota resilience in horse athletes following holidays out to pasture. Scientific reports. [Online] 11 (1).	Meyer, M. M. & Bobeck, E. A. (2023) Undergraduate student attitudes to current poultry industry issues over four semesters: surveying an introductory poultry science course. Journal of animal science. [Online] 101.
Mach, N., Ruet, A., Clark, A., Bars-Cortina, D., Ramayo-Caldas, Y., Crisci, E., Pennarun, S., Dhorne-Pollet, S., Foury, A., Moisan, M.P. and Lansade, L., 2020. Priming for welfare: gut microbiota is associated with equitation conditions and behavior in horse athletes. Scientific reports, 10(1), pp. 1–19.	Miao, Z. et al. (2020) Current Societal Views about Sustainable Wildlife Management and Conservation: A Survey of College Students in China. Animals: an open access journal from MDPI. [Online] 10 (10).
Malinowski, K. et al. (2018) The Effects of Equine Assisted Therapy on Plasma Cortisol and Oxytocin Concentrations and Heart Rate Variability in Horses and Measures of Symptoms of Post-Traumatic Stress Disorder in Veterans. Journal of equine veterinary science. [Online] 6417–26.	Moggy, M. A. et al. (2017) Management practices associated with stress in cattle on western Canadian cow–calf operations: A mixed methods study1. Journal of animal science. [Online] 95 (4), 1836–1844.
Marliani, G. et al. (2021) Evaluation of Horses’ Daytime Activity Budget in a Model of Ethological Stable: A Case Study in Italy. Journal of applied animal welfare science. [Online] 24 (2), 200–213.	Mulder, M. & Zomer, S. (2017) Dutch Consumers’ Willingness to Pay for Broiler Welfare. Journal of applied animal welfare science. [Online] 20 (2), 137–154.
** Mattila-Rautiainen, S., Venojärvi, M., Rautiainen, H. and Keski-Valkama, A., 2023. The impact on physical performance, pain and psychological wellbeing of chronic low back pain patients during 12-weeks of equine-facilitated therapy intervention. Frontiers in Veterinary Science, 10.	Muldoon, J.C. and Williams, J.M., 2021. The challenges and future development of animal welfare education in the UK. Animal Welfare, 30(2), pp. 197–209.
** McKendree, M. G. S. et al. (2014) BIOETHICS SYMPOSIUM II: Current factors influencing perceptions of animals and their welfare1. Journal of animal science. [Online] 92 (5), 1821–1831.	Munoz, C. A. et al. (2022) Improving Communication in the Red Meat Industry: Opinion Leaders May Be Used to Inform the Public About Farm Practices and Their Animal Welfare Implications. Frontiers in Psychology. [Online] 13.
McLean, A.N. and McGreevy, P.D., 2010. Horse-training techniques that may defy the principles of learning theory and compromise welfare. Journal of Veterinary Behavior, 5(4), pp. 187–195.	Murphy, B. et al. (2022) A Qualitative Exploration of Challenges and Opportunities for Dog Welfare in Ireland Post COVID-19, as Perceived by Dog Welfare Organisations. Animals: an open access journal from MDPI. [Online] 12 (23).
Mellor, D. & Beausoleil, N. (2017) Equine Welfare during Exercise: An Evaluation of Breathing, Breathlessness and Bridles. Animals: an open access journal from MDPI. [Online] 7 (6).	Nadlučnik, E. et al. (2022) Discrepancies between farmers’ perceptions and actual animal welfare conditions on commercial pig farms. Frontiers in veterinary science. [Online] 9.
Mellor, D. J. (2020) Mouth Pain in Horses: Physiological Foundations, Behavioural Indices, Welfare Implications, and a Suggested Solution. Animals: an open access journal from MDPI. [Online] 10 (4).	Neff, R. A. et al. (2018) Reducing meat consumption in the USA: a nationally representative survey of attitudes and behaviours. Public health nutrition. [Online] 21 (10), 1835–1844.
Mendonça, T., Bienboire-Frosini, C., Kowalczyk, I., Leclercq, J., Arroub, S. and Pageat, P., 2019. Equine activities influence horses’ responses to different stimuli: Could this have an impact on equine welfare?. Animals, 9(6), p. 290.	Nijland, H. et al. (2018) Exploring the Framing of Animal Farming and Meat Consumption: On the Diversity of Topics Used and Qualitative Patterns in Selected Demographic Contexts. Animals: an open access journal from MDPI. [Online] 8 (2).
Mercer-Bowyer, S. (2017) Use of fecal glucocorticoid and salivary cortisol concentrations as a measure of well-being of New York City carriage horses. Journal of the American Veterinary Medical Association. 250 (3), 316–321.	Nolan, H. R. J. et al. (2022) A Cage Is a Cage, Unless You Educate. Rhetoric Negatively Impacts Support for a Novel Housing System for Laying Hens Unless the Public Are Educated. Frontiers in veterinary science. [Online] 9.
Merkies, K. and Franzin, O., 2021. Enhanced understanding of horse–human interactions to optimize welfare. Animals, 11(5), p. 1347.	Palmer, A. et al. (2021) Getting to grips with wildlife research by citizen scientists: What role for regulation? People and nature. [Online] 3 (1), 4–16.
Mullan, S. et al. (2014) The welfare of long-line tethered and free-ranging horses kept on public grazing land in South Wales. Animal welfare. [Online] 23 (1), 25–37.	Pfeiffer, J. et al. (2021) Understanding the public attitudinal acceptance of digital farming technologies: a nationwide survey in Germany. Agriculture and human values. [Online] 38 (1), 107–128.
Munsters, C. C. B. M. et al. (2013) A prospective study on a cohort of horses and ponies selected for participation in the European Eventing Championship: reasons for withdrawal and predictive value of fitness tests. BMC veterinary research. [Online] 9 (1).	* Pickering, P. & Hockenhull, J. (2020) Optimising the Efficacy of Equine Welfare Communications: Do Equine Stakeholders Differ in Their Information-Seeking Behaviour and Communication Preferences? Animals: an open access journal from MDPI. [Online] 10 (1).
Noble, G.K., 2023. Horse Husbandry–Nutrition, Management and Welfare. Animals, 13(1), p. 169.	Place, S. E. & Mitloehner, F. M. (2014) The Nexus of Environmental Quality and Livestock Welfare. Annual review of animal biosciences. [Online] 2 (1), 555–569.
Nyberg, K. et al. (2011) Treatment with Ca(OH)2 for inactivation of Salmonella Typhimurium and Enterococcus faecalis in soil contaminated with infected horse manure. Journal of applied microbiology. [Online] 110 (6), 1515–1523.	Reuber, H. & Muschalla, B. (2022) Dietary identity and embitterment among vegans, vegetarians and omnivores. Health psychology and behavioral medicine. [Online] 10 (1), 1038–1055.
Oesch, S. et al. (2022) Owner reported clinical signs and treatment decisions in equine pastern dermatitis. Schweiz Arch Tierheilkd. [Online] 164 (5), 401–412.	Riggio, G. et al. (2019) Feeding Enrichment in a Captive Pack of European Wolves (Canis Lupus Lupus): Assessing the Effects on Welfare and on a Zoo’s Recreational, Educational and Conservational Role. Animals: an open access journal from MDPI. [Online] 9 (6).
Pickering, P. & Hockenhull, J. (2020) Optimising the Efficacy of Equine Welfare Communications: Do Equine Stakeholders Differ in Their Information-Seeking Behaviour and Communication Preferences? Animals: an open access journal from MDPI. [Online] 10 (1).	Rioja-Lang, F. C. et al. (2020) Prioritization of Farm Animal Welfare Issues Using Expert Consensus. Frontiers in veterinary science. [Online] 6.
Radkowska, I. (2020) Stereotypic behaviour in cattle, pigs and horses—a review. Animal science papers and reports/. 38 (4), 303–319.	* Rioja-Lang, F. et al. (2020) Prioritisation of animal welfare issues in the UK using expert consensus. The Veterinary record. [Online] 187 (12), 490–490.
Raspa, F. et al. (2022) A high-starch vs. high-fibre diet: effects on the gut environment of the different intestinal compartments of the horse digestive tract. BMC veterinary research. [Online] 18 (1).	Ritter, C. et al. (2021) Views of American animal and dairy science students on the future of dairy farms and public expectations for dairy cattle care: A focus group study. Journal of dairy science. [Online] 104 (7), 7984–7995.
Rioja-Lang, F. et al. (2020) Prioritisation of animal welfare issues in the UK using expert consensus. The Veterinary record. [Online] 187 (12), 490–490.	Robbins, J. et al. (2015) Stakeholder views on treating pain due to dehorning dairy calves. Animal welfare. [Online] 24 (4), 399–406.
Rochais, C., Henry, S. and Hausberger, M., 2018. “Hay-bags” and “Slow feeders”: Testing their impact on horse behaviour and welfare. Applied Animal Behaviour Science, 198, pp. 52–59.	Ronto, R. et al. (2016) Adolescents’ perspectives on food literacy and its impact on their dietary behaviours. Appetite. [Online] 107549–557.
Rørvang, M. V. et al. (2020) Sensory Abilities of Horses and Their Importance for Equitation Science. Frontiers in veterinary science. [Online] 7.	Russell, E. R. et al. (2022) Views of Western Canadian dairy producers on calf rearing: An interview-based study. Journal of dairy science. [Online] 105 (2), 1480–1492.
Rowland, M. (2019) A Study of Traveller Horse-owners’ Attitudes to Horse Care and Welfare Using an Equine Body Condition Scoring System. Animals: an open access journal from MDPI. [Online] 9 (4).	Ruzgys, S. & Pickering, G. J. (2020) Perceptions of Cultured Meat Among Youth and Messaging Strategies. Frontiers in sustainable food systems. [Online] 4.
Ruet, A. et al. (2019) Housing Horses in Individual Boxes Is a Challenge with Regard to Welfare. Animals: an open access journal from MDPI. [Online] 9 (9).	Sadegholvad, S. et al. (2017) What Should Be Taught in Secondary Schools’ Nutrition and Food Systems Education? Views from Prominent Food-Related Professionals in Australia. Nutrients. [Online] 9 (11).
Ruet, A. et al. (2020) Effects of a temporary period on pasture on the welfare state of horses housed in individual boxes. Applied animal behaviour science. [Online] 228.	Sane, R. (2021) Understanding the nature and dimensions of litigation crowdfunding: A visual analytics approach. PloS one. [Online] 16 (4).
Sarrafchi, A. & Blokhuis, H. J. (2013) Equine stereotypic behaviors: Causation, occurrence, and prevention. Journal of veterinary behavior: clinical applications and research: official journal of: Australian Veterinary Behaviour Interest Group, International Working Dog Breeding Association. [Online] 8 (5), 386–394.	Sans, P. & Sanjuán-López, A. I. (2015) Beef animal welfare, attitudes and Willingness to Pay: A regional comparison across the Pyrenees. Spanish journal of agricultural research. [Online] 13 (3).
Sato, S., 2016. Applied animal behaviour science in Japan and the culture of ‘aigo’. In *Animals and us: 50 years and more of applied ethology* (pp. 12–37). Wageningen Academic Publishers.	Saraceni, J. et al. (2022) Ontario Dairy Producers’ Perceived Barriers and Motivations to the Use of Pain Control for Disbudding and Dehorning Calves: A Qualitative Study. Animals: an open access journal from MDPI. [Online] 12 (8).
Schuurman, N., 2015. Conceptions of equine welfare in Finnish horse magazines. Society & Animals, 23(3), pp. 250–268.	Sasaki, N. (2022) A New Online Hands-on Practical Training Method Suitable for COVID-19 Social Distancing Regulations. Medical science educator. [Online] 32 (3), 691–695.
Scofield, R.M., Scofield, S. and Briggs, E., 2019. Conspicuity equipment and its contribution to the welfare of horse and rider combinations using the road system in the United Kingdom. Journal of equine veterinary science, 82, p. 102770.	Schiano, A. et al. (2021) Consumer perception of dried dairy ingredients: Healthy, natural, and sustainable? Journal of dairy science. [Online] 104 (12), 12427–12442.
Scopa, C. et al. (2020) Inside the Interaction: Contact With Familiar Humans Modulates Heart Rate Variability in Horses. Frontiers in veterinary science. [Online] 7.	Schirmacher, H., Elshiewy, O. and Boztug, Y., 2023. That’s not natural! Consumer response to disconfirmed expectations about ‘natural’food. Appetite, 180, p. 106270.
Smith, R., Furtado, T., Brigden, C., Pinchbeck, G. and Perkins, E., 2022. A qualitative exploration of UK Leisure Horse-owners’ Perceptions of equine wellbeing. Animals, 12(21), p. 2937.	Shivley, C. et al. (2019) Management of preweaned bull calves on dairy operations in the United States. Journal of dairy science. [Online] 102 (5), 4489–4497.
Solis, C. N. et al. (2018) Sudden death in sport and riding horses during and immediately after exercise: A case series. Equine veterinary journal. [Online] 50 (5), 644–648.	Sinclair, M. & Phillips, C. J. (2019) Asian Livestock Industry Leaders’ Perceptions of the Importance of, and Solutions for, Animal Welfare Issues. Animals: an open access journal from MDPI. [Online] 9 (6).
Sullivan, P. et al. (2022) A Nationwide Survey of Animal Science Students’ Perceptions of Animal Welfare across Different Animal Categories at Institutions in the United States. Animals: an open access journal from MDPI. [Online] 12 (17),	Situmorang, R. O. P. et al. (2022) Purchase Intention on Sustainable products: A Case study on Free-Range Eggs in Taiwan. Applied economics. [Online] 54 (32), 3751–3761.
Tarazona, A. M. et al. (2020) Human Relationships with Domestic and Other Animals: One Health, One Welfare, One Biology. Animals: an open access journal from MDPI. [Online] 10 (1).	Soon, J. M. & Wallace, C. (2017) Application of theory of planned behaviour in purchasing intention and consumption of Halal food. Nutrition and food science. [Online] 47 (5), 635–647.
Thompson, K. and Clarkson, L., 2019. How owners determine if the social and behavioral needs of their horses are being met: Findings from an Australian online survey. Journal of Veterinary Behavior, 29, pp. 128–133.	Specht, K. et al. (2019) How Will We Eat and Produce in the Cities of the Future? From Edible Insects to Vertical Farming—A Study on the Perception and Acceptability of New Approaches. Sustainability. [Online] 11 (16).
Thompson, K. and Haigh, L., 2018. Perceptions of Equitation Science revealed in an online forum: Improving equine health and welfare by communicating science to equestrians and equestrian to scientists. Journal of veterinary behavior, 25, pp. 1–8.	Spooner, J. M. et al. (2014) Attitudes of Canadian citizens toward farm animal welfare: A qualitative study. Livestock science. [Online] 163150–158.
Tuomola, K. et al. (2021) Bit-Related Lesions in Event Horses After a Cross-Country Test. Frontiers in veterinary science. [Online] 8.	Spooner, S. L. & Stride, J. R. (2021) Animal-human two-shot images: Their out-of-context interpretation and the implications for zoo and conservation settings. Zoo biology. [Online] 40 (6), 563–574.
Uldahl, M., Christensen, J.W. and Clayton, H.M., 2021. Relationships between the Rider’s pelvic mobility and balance on a gymnastic ball with equestrian skills and effects on horse welfare. Animals, 11(2), p. 453.	Spooner, S. L. et al. (2021) Evaluating the effectiveness of live animal shows at delivering information to zoo audiences. International journal of science education. [Online] 11 (1), 1–16.
Visser, E. K. & Van Wijk-Jansen, E. E. (2012) Diversity in horse enthusiasts with respect to horse welfare: An explorative study. Journal of veterinary behavior: clinical applications and research: official journal of: Australian Veterinary Behaviour Interest Group, International Working Dog Breeding Association. [Online] 7 (5), 295–304.	Stubbe Solgaard, H. & Yang, Y. (2011) Consumers’ perception of farmed fish and willingness to pay for fish welfare. British Food Journal. [Online] 113 (8–9), 997–1010.
Wade, C. (2016) Current Welfare Problems Facing Horses in Great Britain as Identified by Equine Stakeholders. PloS one. [Online] 11 (8).	Tickle, L., Von Essen, E. and Fischer, A., 2022. Expanding arenas for learning hunting ethics, their grammars and dilemmas: An examination of young hunters’ enculturation into modern hunting. Sociologia Ruralis, 62(3), pp. 632–650.
Ward, A.B., Stephen, K., Argo, C.M., Harris, P.A., Watson, C.A., Neacsu, M., Russell, W., Grove-White, D.H. and Morrison, P.K., 2021. COVID-19 impacts equine welfare: Policy implications for laminitis and obesity. Plos one, 16(5), p. e0252340.	Toma, L. et al. (2012) Consumers and animal welfare. A comparison between European Union countries. Appetite. [Online] 58 (2), 597–607.
Warren-Smith, A. K. (2007) The use of blended positive and negative reinforcement in shaping the halt response of horses (*Equus caballus*). Animal welfare. 16 (4), 481–488.	Väärikkälä, S. et al. (2019) Assessment of Welfare Problems in Finnish Cattle and Pig Farms Based on Official Inspection Reports. Animals: an open access journal from MDPI. [Online] 9 (5).
Watson, E. et al. (2020) Characterization of Horse Use in Therapeutic Horseback Riding Programs in the United States: A Pilot Survey. Journal of equine veterinary science. [Online] 92.	Vanhonacker, F. et al. (2009) Societal concern related to stocking density, pen size and group size in farm animal production. Livestock science. [Online] 123 (1), 16–22.
Wilkins, A. M. et al. (2015) Factors affecting the Human Attribution of Emotions toward Animals. Anthrozoös. [Online] 28 (3), 357–369.	Vargas-Bello-Pérez, E. et al. (2022) Consumer attitudes toward dairy products from sheep and goats: A cross-continental perspective. Journal of dairy science. [Online] 105 (11), 8718–8733.
Williams, B. et al. (2022) What is equine hoarding and can ‘motivational interviewing’ training be implemented to help enable behavioural change in animal owners? Equine veterinary education. [Online] 34 (1), 29–36.	* Visser, E. K. & Van Wijk-Jansen, E. E. (2012) Diversity in horse enthusiasts with respect to horse welfare: An explorative study. Journal of veterinary behavior: clinical applications and research: official journal of: Australian Veterinary Behaviour Interest Group, International Working Dog Breeding Association. [Online] 7 (5), 295–304.
Zebisch, A. et al. (2014) Effect of different head-neck positions on physical and psychological stress parameters in the ridden horse. Journal of animal physiology and animal nutrition. [Online] 98 (5), 901–907.	Wang, O. & Scrimgeour, F. (2023) Consumer segmentation and motives for choice of cultured meat in two Chinese cities: Shanghai and Chengdu. British Food Journal. [Online] 125 (2), 396–414.
	Weary, D. M. et al. (2011) Tail docking dairy cattle: Responses from an online engagement1. Journal of animal science. [Online] 89 (11), 3831–3837.
	Whitehouse-Tedd, K. M. et al. (2022) Assessing the Visitor and Animal Outcomes of a Zoo Encounter and Guided Tour Program with Ambassador Cheetahs. Anthrozoös. [Online] 35 (2), 307–322.
	Williams, J.M., Cardoso, M.P., Zumaglini, S., Finney, A.L., Scottish SPCA and Knoll, M.A., 2022. “Rabbit Rescuers”: A school-based animal welfare education intervention for young children. Anthrozoös, 35(1), pp. 55–73.
	Wold, T. (2023) Letters to the Public: What Goes Viral Online? SAGE open. [Online] 13 (1).
	Worsley, A. et al. (2015) Food concerns and support for environmental food policies and purchasing. Appetite. [Online] 9148–55.
	Xu, L. (2019) Consumers’ Willingness to Pay for Food with Information on Animal Welfare, Lean Meat Essence Detection, and Traceability. International journal of environmental research and public health. [Online] 16 (19).
	Yang, Y.-C. & Hong, C.-Y. (2019) Taiwanese Consumers’ Willingness to Pay for Broiler Welfare Improvement. Animals: an open access journal from MDPI. [Online] 9 (5).
	Yildirim, A. (2018) Perception of animal sentience by Brazilian and French citizens: The case of sheep welfare and sentience. PloS one. [Online] 13 (7).
	Yildirim, A. (2021) Consumer perceptions of antimicrobial use in animal husbandry: A scoping review. PloS one. [Online] 16 (12).
	Zanoli, R. et al. (2013) Organic label as an identifier of environmentally related quality: A consumer choice experiment on beef in Italy. Renewable agriculture and food systems. [Online] 28 (1), 70–79.
	Zhang, L. et al. (2008) Wildlife trade, consumption and conservation awareness in southwest China. Biodiversity and conservation. [Online] 17 (6), 1493–1516.
